# Liver histopathology in dogs with naturally acquired *Babesia rossi* infection

**DOI:** 10.3389/fvets.2026.1765994

**Published:** 2026-06-08

**Authors:** Heidi Horrell, Andrew Leisewitz, Sarah Clift

**Affiliations:** 1 Alma Veterinary Hospital, Smoothfield, United Kingdom; 2 Department of Clinical Sciences, Auburn University College of Veterinary Medicine, Auburn, AL, United States; 3 Faculty of Veterinary Science, Department of Companion Animal Clinical Services, University of Pretoria, Onderstepoort, South Africa; 4 Faculty of Veterinary Science, Department of Anatomy and Physiology, University of Pretoria, Onderstepoort, South Africa

**Keywords:** babesiosis, canine, hepatic, hypoxia, Kupffer cells, macrophages

## Abstract

**Introduction:**

In sub-Saharan Africa, canine babesiosis, the most significant tick-borne disease in dogs, is primarily caused by the highly virulent intra-erythrocytic protozoan *Babesia rossi,* transmitted by ixodid ticks. The disease presents in two forms: uncomplicated and complicated. The former is characterized by hemolysis, while the latter involves severe systemic inflammation and multiple organ dysfunction, leading to high morbidity and mortality. Building on recent evidence of severe immunoinflammatory disruption in the spleen, lung, and bone marrow, this study examines liver histopathology in fatal *Babesia rossi* infection to determine whether the frequent hyperbilirubinemia and icterus of complicated babesiosis reflect primary hepatic injury or secondary systemic effects.

**Methods:**

Liver samples from 10 dogs that succumbed to *Babesia rossi* infection were compared with samples from four healthy control dogs.

**Results:**

Significant hematological changes included reduced red cell count, hematocrit, and platelet concentration, while biochemical alterations showed increased serum ALT and urea. Key histopathological findings in the livers of *Babesia*-infected dogs showed severe diffuse dilation of the spaces of Disse, cholestasis, and hypertrophy of Kupffer cells containing bile and hemosiderin pigments. Additional findings included erythropoiesis, congestion, and widespread mononuclear inflammation, predominantly involving monocytes and macrophages with fewer lymphocytes and plasma cells, present in both intravascular and interstitial areas. Centrilobular hepatocytes exhibited coagulative necrosis or prominent hydropic vacuolation, likely attributable to severe hemolysis and resulting hypoxia, similar to findings reported in human malaria and sepsis.

**Discussion:**

These observations improve understanding of the pathomechanisms underlying fatal babesiosis in dogs, particularly its effects on the liver, and may help refine targeted treatment strategies.

## Introduction

Canine babesiosis, one of the most important tick-borne diseases in dogs, is caused by *Babesia* parasites, protozoans of the *Piroplasmida* family that infect red blood cells and are transmitted by ixodid ticks (1). Mortality varies significantly, from 1% in *B. vogelli* infections to up to 45% in *B. rossi* infections (1). Dogs may also act as asymptomatic carriers, potentially transmitting the infection (2). Mature *Babesia* sporozoites from the tick’s saliva enter the vertebrate host’s erythrocytes after 2–3 days of tick attachment. Sporozoites then differentiate into trophozoites (1). These trophozoites then proliferate through merogony, producing merozoites that repeatedly infect new erythrocytes, perpetuating the infection cycle. The disease exhibits seasonal prevalence, occurring more commonly during warm, humid months when tick activity increases (1).

*Babesia* infection causes progressive hemolytic anemia, with clinical signs primarily due to hemolysis (3), ranging from mild and even subclinical forms to severe, often peracute forms typically associated with high mortality. Disease severity varies based on host factors such as the animal’s age, immune status, splenectomy history, and concurrent infections (2, 3), with younger dogs more prone to immune-mediated hemolysis and older dogs typically developing severe systemic inflammation (4, 5). This may well be because the adult immune system is more primed than the juvenile system, as has been shown for human malaria (6). The incubation period ranges from 4–21 days (1, 3). Babesiosis caused by *B. rossi* presents in two forms: Uncomplicated and complicated. Uncomplicated cases are presented with signs such as lethargy, weakness, fever, mild to moderate hemolytic anemia, splenomegaly (from extravascular hemolysis), tachycardia, tachypnoea, anorexia, vomiting, and thrombocytopenia, without organ dysfunction (1, 3). In more severe cases, signs such as icterus, respiratory distress and collapse may be observed (1, 7). Complicated babesiosis however, involves not only hemolysis and tissue hypoxia but also an excessive immune response to infection, leading to systemic inflammation and the multiple organ dysfunction syndrome (MODS), which is akin to a septic shock like syndrome (8–10).

Hemolysis in babesiosis is attributed to three basic mechanisms: Membrane damage caused by merozoites as they exit red blood cells, macrophage-mediated erythrophagocytosis in the spleen, liver, and bone marrow, and immune activation that triggers hemolysis through anti-erythrocyte antibodies and complement cascade activation (9). The degree of anemia does not correlate directly with parasitemia or hemolysis, indicating additional contributing factors such as erythrocyte sludging in peripheral capillaries, oxidative damage to erythrocytes, or an inadequate regenerative response to severe anemia (10). In addition, the resulting anemic hypoxia may promote reversible or irreversible hepatocellular injury and cholestasis, which contribute to hyperbilirubinemia and are reflected clinically by increases in alanine aminotransferase (ALT) and alkaline phosphatase (ALP) (11).

Despite the clinical importance of *B. rossi* in dogs in sub-Saharan Africa, detailed pathology of this infection has only recently begun to be characterized. Studies using the same cohort of naturally infected dogs have described marked lesions in the spleen, lung, and bone marrow (12–14), demonstrating widespread multi-organ involvement with pronounced monocyte–macrophage activation and redistribution. These findings highlight the need for a coordinated, organ-level understanding of fatal *B. rossi* infection, especially in light of the well-documented hematologic and biochemical abnormalities.

Hepatic involvement in hemoparasitic disease is well described in other species and consistently reflects the effects of hemolysis, hypoxia, microvascular injury, and systemic inflammation. In dogs, *B. gibsoni* and *B. canis* infections cause diffuse mononuclear-rich hepatitis, Kupffer cell hypertrophy with hemosiderosis and erythrophagocytosis, sinusoidal congestion, cholestasis, and centrilobular hepatocellular degeneration or necrosis (15–17). Lipid degeneration of hepatocytes and bile pigment in hepatocytes have been described in *Babesia canis*-infected dogs (18). Comparable lesions occur in cattle infected with *B. bovis* or *B. bigemina,* namely hepatomegaly, cholestasis, Kupffer cell activation, hemosiderosis, mononuclear leukostasis, and centrilobular injury (19–21), and in sheep, gerbils, rats, horses, and cats with various *Babesia* species, which show overlapping hepatocellular swelling or necrosis, cholestasis, Kupffer cell hyperplasia, sinusoidal congestion, extramedullary hematopoiesis (EMH), and lymphoplasmacytic inflammation (22–28). Similar patterns occur in *Plasmodium falciparum* malaria, where hemolysis-associated hypoxia, cytoadherence and sequestration-related microcirculatory dysfunction, and a cytokine-driven inflammatory response produce hepatomegaly, Kupffer cell hyperplasia with hemozoin deposition, sinusoidal congestion, cholestasis, and variable hepatocellular necrosis (29–32).

However, despite these well-established hepatic lesions in related infections, the liver pathology of *B. rossi* infection in dogs has not been described. This gap is clinically relevant because many dogs with severe *B. rossi* infection show biochemical evidence of hepatic dysfunction (33, 34), and the disease is characterized by anemia-induced hypoxia, hemolysis-associated oxidative stress, inflammatory mediator release, and microcirculatory impairment, all mechanisms capable of producing significant hepatic injury (4, 9, 34–36). A detailed assessment of the liver is therefore needed to clarify whether hepatic lesions play a direct role in icterus and organ dysfunction or primarily mirror systemic pathology.

In this study, as part of a broader multi-organ investigation of fatal *B. rossi* infection in dogs, we describe the histopathologic and immunohistochemical changes in the liver, addressing an important gap in current knowledge. Special stains and markers for mononuclear leukocytes, chosen to match those used previously in the spleen (12), lung (13), and bone marrow (14) from the same cohort of dogs, were included to extract as much information as possible from infected and control livers to help clarify the underlying pathophysiology of this complex multisystem disease.

## Materials and methods

### Experimental design

This was a semi quantitative, retrospective, case controlled descriptive study. Liver specimens from dogs naturally infected with *Babesia rossi,* that died (n = 7) or were euthanized (n = 3) due to a very grave prognosis at the Onderstepoort Veterinary Academic Hospital (OVAH), were obtained from an existing tissue bank maintained by a co-investigator (AL) (34). Euthanasia was performed by means of pentobarbitone overdose administered intravenously (100 mg/kg body weight). The infected canine cohort comprised 10 dogs, including one female and nine males, with diverse ages (mean = 52.8 months, median = 50 months) and weights (mean = 28.9 kg, median = 34 kg). The group represented a mix of breeds: Boerboel, German Shepherd, Great Dane, Cocker Spaniel, Boxer, Rottweiler, Pitbull terrier and Africanis.

*Babesia rossi* infection was diagnosed on Diff-Quik-stained peripheral capillary blood smears and a mono-infection was subsequently confirmed using polymerase chain reaction (PCR) and reverse-line blot (RLB) hybridization assays on ethylenediaminetetraacetic acid (EDTA) blood samples (37). Serum and EDTA blood samples were collected from these dogs prior to the administration of any treatment. These dogs either died pre-treatment, post-treatment, or were euthanized due to a poor prognosis. Their liver samples were histologically and immunohistochemically compared with liver samples from four healthy control dogs, which were euthanized for welfare reasons (homeless dogs not suitable for adoption) and collected prospectively. The control group comprised three females and one male dog, with diverse ages (mean = 18.3 months, median = 15.5 months), weights (mean = 13.6 kg, median = 12.5 kg), and breeds (Pitbull terrier, Africanis, and Boerboel). These dogs were clinically healthy individuals routinely euthanized because they were not suitable for adoption. Health status was confirmed based on normal habitus, physiological parameters (rectal temperature, heart rate, respiratory rate, mucous membrane color, capillary refill time, pulse quality, hydration status), unremarkable hematology and serum biochemistry results including pre- and post- bile acid determinations, and the absence of hemoparasites, verified by PCR-RLB testing of EDTA-preserved blood samples (37).

Dogs were excluded from the study if they tested PCR-positive for hemoparasites other than *B. rossi,* including *B. vogeli* and *Theileria* spp., or for rickettsial bacteria such as *Ehrlichia canis, and Anaplasma* spp. Additional exclusion criteria included the administration of anti-inflammatory medications within 4 weeks prior to presentation, the presence of co-morbidities identified during macroscopic postmortem examination, or after detailed histological evaluation. Ethical approval for this study was granted by the University of Pretoria Animal Ethics committee (REC040-21).

### Sample collection

Liver samples for histopathology were collected from *B. rossi*-infected and control dogs within one hour post-euthanasia, following the protocol outlined by Leisewitz et al. (7). A 1cm^3^ sample was taken from each liver lobe, fixed in 10% neutral buffered formalin for up to 5 days, then trimmed and embedded in paraffin.

### Histopathology

Formalin-fixed, paraffin-embedded liver specimens were sectioned at 4-5μm and stained with hematoxylin and eosin (H&E) following standard operating procedures in the histopathology laboratory of the Department of Paraclinical Sciences (DPS), Faculty of Veterinary Sciences (FVS), University of Pretoria (UP). The sections were examined using an Olympus CX21 light microscope. For control dogs, the liver specimen with the best tissue preservation was selected for further special stains and immunohistochemical (IHC) analysis. In the *Babesia*-infected dogs, the initial histological analysis showed a consistent distribution of lesions across all liver lobes. Because of this specimens selected for further special stains and IHC were those with good preservation, and with the least artifacts.

Special stains included Gordon and Sweet’s silver (GSS) method for reticular fibres, to assess whether hepatic architecture was disrupted in areas of necrosis; Perl’s Prussian blue for ferric iron, detecting iron storage complexes (ferritin and hemosiderin) resulting from hemolysis; Martius Scarlet Blue (MSB) for fibrin, to identify deposition associated with systemic inflammation and potential disseminated intravascular coagulation (DIC); and Luxol fast blue (LFB), normally used for myelin, to highlight intraerythocytic *Babesia* parasites in tissue sections (38). All staining procedures were performed according to internal SOPs adapted from Bancroft’s methods (39).

### Immunohistochemistry

Routine chromogen-based indirect immunoperoxidase staining was performed by hand, according to optimized protocols in the IHC laboratory, DPS, FVS, UP (12). The targeted cellular antigens included CD3 (T-lymphocytes), CD20 (B-lymphocytes and, to a lesser extent, plasma cells), CD204 (macrophages), Iba-1 (activated monocytes, macrophages and dendritic cells), MAC387 (neutrophils, dendritic cells, monocytes, activated macrophages), and MUM1/IRF-4 (mature B-lymphocytes and plasma cells). Antibody species specificity has been documented in previous studies and recorded by manufacturers (12, 13). The antibody specifications and associated protocols are listed in Table 1.

**Table 1:** Antibody specifications and methodological details for immunohistochemistry.

Antigen	Host	Clone	Manufacturer	Antigen retrieval	Dilution (incubation time)
CD3	Rabbit	AO452	Dako	HIER; Citrate buffer; pH 6.0	1:600 (1 h)
CD20	Rabbit	PA5-16701	Thermo Fisher Scientific	HIER; Tris-EDTA buffer; pH 9.0	1:800 (2 h)
CD204	Mouse	SRA-E5	Abnova	HIER; Tris-EDTA buffer; pH 9.0	1:400 (1 h)
Iba-1	Rabbit	Ab178846	Abcam	HIER; Citrate buffer; pH 6.0	1:1600 (2 h)
Myeloid/histiocyte antigen	Mouse	M0747	Dako	HIER; Tris-EDTA buffer; pH 9.0	1:400 (1 h)
MUM1/IRF4	Mouse	M7259	Dako	HIER; Tris-EDTA buffer; pH 9.0	1:50 (1 h)

HIER, heat-induced epitope retrieval.

Tissue sections from the control and *Babesia-*infected dogs were mounted on positively charged Superfrost glass slides and heated at 40 °C overnight. Thereafter, slides were routinely dewaxed in xylene and rehydrated through graded ethanol, followed by rinsing in distilled water. In order to quench endogenous peroxidase activity, the slides were incubated with 3% hydrogen peroxide in methanol for 15 min at room temperature. Heat-induced epitope retrieval was performed in a microwave oven at 96 °C, either in citrate buffer (pH = 6.0) or Tris-EDTA buffer (pH = 9.0), for 20 min (Table 1). Slides were allowed to cool on the bench, after which they were rinsed in distilled water. This was followed by rinsing in 0.1 molar (M) phosphate buffered saline (PBS; pH = 7.6), which contained 0.1% bovine serum albumin (BSA). Slides were then incubated with the primary antibodies at room temperature (12, 13). After rinsing in distilled water, followed by PBS-BSA buffer, the slides were treated with a polymer-based detection method (BioGenex Super Sensitive Polymer-HRP IHC detection system, BioGenex, Fremont, USA), according to manufacturer’s instructions. After rinsing in distilled water, followed by PBS-BSA buffer, immunoreactivity was visualized using a 3,3′- diaminobenzidine (DAB) chromogen. Slides were counterstained with Mayer’s hematoxylin for 20 s, rinsed under tap water for 10 min, routinely dehydrated in ascending graded ethanols, cleared in xylene, mounted with entellan (Thermo Scientific) and coverslipped. Reactive dog lymph node and spleen samples served as positive tissue controls, while buffer substituting the primary antibody was used for negative reagent control purposes to assess reaction specificity. Positive staining was brown. CD3-specific immunoreactivity was cytoplasmic and membranous, CD20-specific staining was membranous, CD204 and Iba-1 immunoreactivity was both cytoplasmic and membranous, and MAC387 positivity was both nuclear and cytoplasmic, while MUM1/IRF-4 immunoreactivity was predominantly nuclear.

### Data analysis

The study was mainly descriptive, incorporating histopathology, special stains and immunohistochemical scoring to generate semi-quantitative data. Using detailed score sheets (see Supplementary material S1), livers from *Babesia*-infected dogs were compared to those of control dogs. Changes were identified, described, and quantified across the entire liver specimen and within specific zones (centrilobular, midzonal and periportal) of the hepatic lobule.

For IHC scoring, positively stained cells were counted manually on photomicrographs (20x objective; 200x magnification). Counts were performed in three separate high-power fields (HPFs) for each of the three zones per liver specimen per case. A median cell count for each zone was calculated, and the highest and lowest median counts across all zones for each cell phenotype were reported. This provided a range for the median count for each cell type across the three zones.

Statistical analysis was conducted on numerical data using non-parametric tests (Mann Whitney U) to compare medians between groups, owing to the small sample size. This analysis was performed with SPSS, version 24, IBM, with significance set at *p* < 0.05.

## Results

### Hematology

The most significant hematological alterations observed in the *B. rossi*-infected dogs were reductions in red cell count (median 2.1 (standard deviation 1.6) x10^12^/L), hematocrit (median 0.2 (standard deviation 0.1) (L/L)) and platelet concentration (median 40.5 (standard deviation 33.2) x 10^9^/L).

### Biochemistry

The results of the liver-specific serum biochemistry values are summarized in Table 2. The most significant biochemical alterations were increases in ALT and urea.

**Table 2:** Summary of liver-specific serum biochemistry results for control and *Babesia rossi*-infected cases.

Biochemistry parameter	Laboratory reference range	Control median (25th-75th percentile)	*Babesia rossi*- median (25th-75th percentile)	*p*-value* (Control vs. infected cases)
ALP (U/L)	20–165	80.5 (35–120)	119 (90.3–226)	0.142
ALT (U/L)	9–73	21.4 (12.2–25.5)	86.5 (43.3–341.8)	**0.002**
Bile acids (μmol/L)	0–8	2.5 (2.5–3.8)	22 (6–27)	0.109
Urea (mmol/L)	2.3–8.9	3.1 (2.7–4.1)	28.3 (19.4–63.9)	**0.048**
Albumin (g/L)	28–41	33.3 (22.3–36.5)	19.6 (15.7–23.1)	0.054
Total serum protein (TSP) (g/L)	56–73	51.3 (47.6–58.7)	53.7 (46.7–68.9)	0.839
**Bilirubin (μmol/L)	1–6.8	Not measured	83.3 (13.9–173)	Not performed
***Glucose (mmol/L)	3.3–5.5	Not measured	3.6 (2.3–9.4)	Not performed.

The laboratory-specific reference range, median and range of both the control (*n* = 4) and *Babesia rossi*-infected cases (*n* = 10) are listed below with the *P*-value comparing the control and infected cases.

*Significant *P*-values (*P*-value <0.05) are in bold. ** Bilirubin values of the *Babesia rossi*-infected cases were severely elevated compared to the normal laboratory reference range. *** Glucose values of the *Babesia rossi*-infected cases fell mostly within the laboratory reference range.

### Histopathology overview

**Figure 1:**
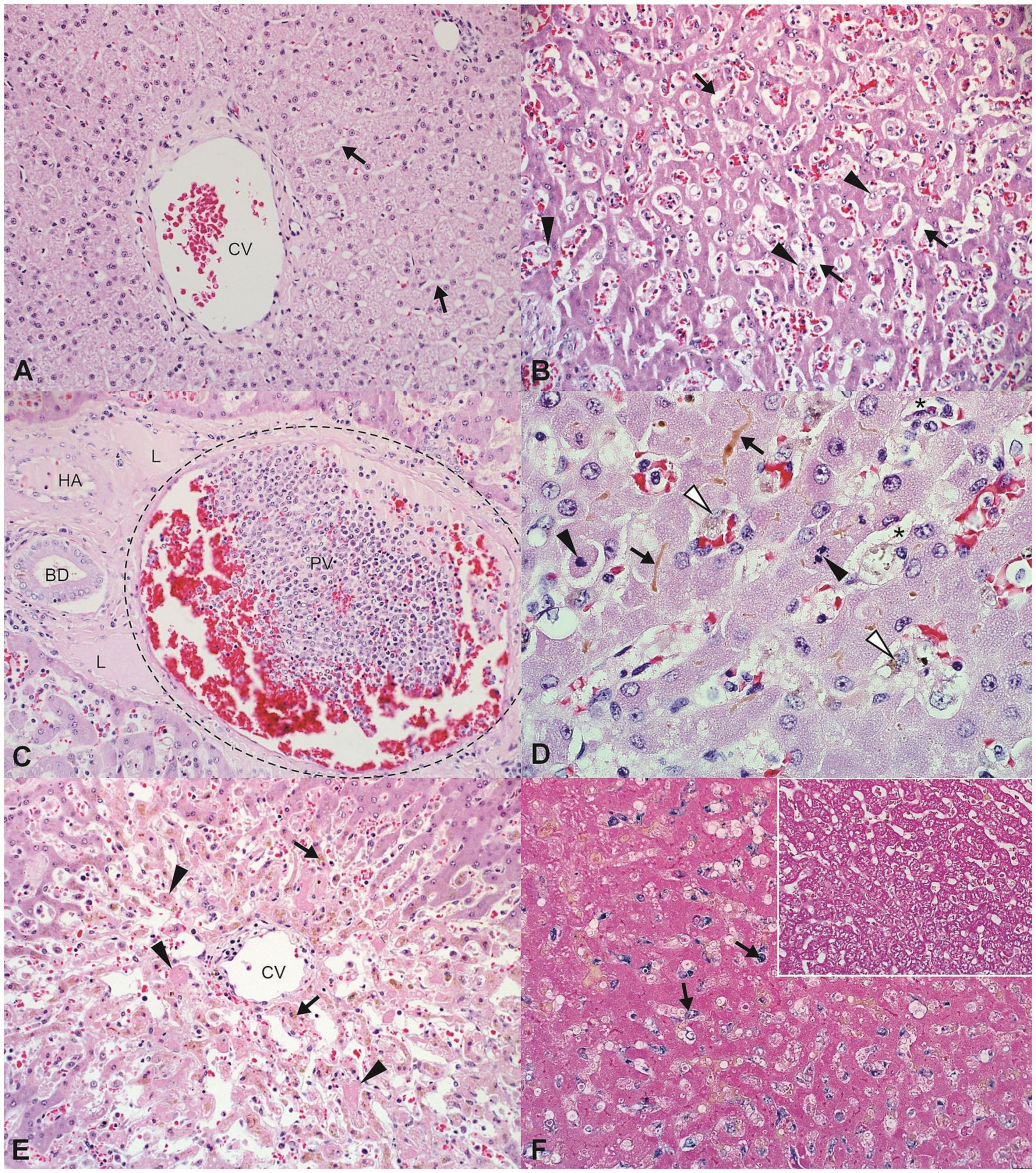
Hepatic histopathology in *Babesia rossi*-infected dogs. Liver sections stained with hematoxylin and eosin (H&E; **A–E**) and Perls’ Prussian blue **(F)**. **(A)** shows liver tissue from an uninfected control dog; **(B–F)** show representative lesions in *Babesia rossi*-infected dogs. BD, bile duct; CV, central vein; HA, hepatic arteriole; L, lymph capillary; PV, portal vein. **(A)** (Control; centrilobular region). Control dog with normal hepatocyte cords and sinusoids (arrows) surrounding the CV. **(B)** (Infected; midzonal region). Marked widening of the spaces of Disse (arrows) and markedly hypertrophic, hemosiderin-laden Kupffer cells (arrowheads). **(C)** (Infected; portal tract). Dense aggregation of monocytoid mononuclear leukocytes within the PV (dashed oval), with mildly dilated lymph capillaries **(L)**. **(D)** (Infected; midzonal region). Canalicular bile stasis (arrows), mitotic hepatocytes (black arrowheads), hypertrophic hemosiderin-laden Kupffer cells (white arrowheads), and widened spaces of Disse (asterisks). **(E)** (Infected; centrilobular region). Necrotic hepatocytes with hypereosinophilic cytoplasm and occasional nuclear fragments (arrowheads), intracellular bile accumulation (arrows), and scattered lymphocytes and plasma cells around the CV. **(F)** (Infected; Perls’ Prussian blue, midzonal region). Kupffer cells containing ferric iron (arrows). Inset: control liver with no Perls’ staining.

Most changes were distributed diffusely across all hepatic zones, with certain lesions more prominent in specific regions when compared with control livers (Figure 1A). Key diffuse changes included severe distension of the spaces of Disse (Figure 1B), accompanied by consistently dilated portal lymph capillaries, and increased numbers of mononuclear leukocytes within the vasculature (Figure 1C) and perivascular interstitium. Kupffer cells were markedly hypertrophic and frequently contained intracytoplasmic hemosiderin pigment (Figures 1D,F). Multifocal small aggregates and individual metarubricytes were observed in all hepatic zones, occurring within vessels and the perivascular interstitium. Megakaryocytes were however scarce. Mild to marked congestion was present in all zones, and bile pigment was consistently observed within hepatocytes and intercellular canaliculi, most prominently in centrilobular regions (Figures 1D,E). Centrilobular hepatocytes commonly exhibited coagulative necrosis (Figure 1E) or prominent hydropic degeneration. Scattered hepatocytes exhibited prominent vesicular nuclei, occasional mitoses (Figure 1D), and increased binucleation, more evident than in the control livers (Figure 1A).

### Detailed results per liver region

#### Portal tracts and periportal hepatocytes

Compared with controls, periportal regions consistently showed severe distension of the spaces of Disse (100% of cases) (Figure 1C), while dilation of lymphatic capillaries was observed in 90% of cases. In the periportal region, Kupffer cells within the sinusoids were notably hypertrophic and often exhibited hemosiderin accumulation or erythrophagocytosis. Intravascular mononuclear leukocytes were increased in all cases (Figure 1C); however, clear perivascular infiltration was present in only half the cases. Rare intravascular neutrophils were observed in portal tracts and periportal sinusoids. Intra- and occasional perivascular nucleated red blood cells were present in all cases but were prominent in only 30%. Ductal, canalicular and hepatocellular bile pigment was identified in 80% of cases. Hydropic vacuolation of periportal hepatocytes was generally mild (70%) but was severe in 20% of cases, with one case showing minimal vacuolation. Prominent vesicular or euchromatic nuclei were observed in 70% of cases, while mild hepatocyte turnover, indicated by mitoses and increased binucleation, was present in 80% of cases.

#### Midzonal area

Midzonal areas demonstrated marked distension of the spaces of Disse in all cases (100%, Figures 1B,D). Obviously hypertrophic Kupffer cells, often containing hemosiderin, were present in 60% of cases (Figures 1B,D,F). Single and small aggregates of metarubricytes, both intra- and perivascular, were commonly observed in 30% of samples. Sinusoidal congestion was mild to moderate in all cases. Prominent hydropic vacuolation involved 40% of cases. Bile pigment, within hepatocytes and canaliculi, was detected in 50% of cases (Figure 1D). Vesicular/euchromatic hepatocyte nuclei were noticeable in 90% of cases, and moderate hepatocyte turnover, indicated by mitoses and binucleation, was evident in 30% of cases.

#### Centrilobular region

Centrilobular hepatocytes exhibited multifocal to coalescing coagulative necrosis or hydropic vacuolation in 80% of cases (Figure 1E), with severe macrovesicular fatty change observed in 10% of samples. Severe distension of the spaces of Disse and prominent dilation of centrilobular lymphatic capillaries were evident in all cases (100%). Intra- and perivascular mononuclear leukocytes were increased in all cases (Figure 1E). In the majority of cases, only occasional neutrophils were observed within central veins and periacinar sinusoids. Severely hypertrophic, readily identifiable Kupffer cells, often containing hemosiderin, were observed in all cases. Nucleated erythrocytes and rare megakaryocytes occurred in 50% of cases, both intra- and perivascularly. Mild to moderate congestion was present throughout, and canalicular and hepatocellular bile pigment was consistently observed (Figure 1E). Hepatocytes with enlarged vesicular nuclei were seen in all cases, whereas prominent hepatocyte turnover, indicated by mitoses and binucleation, was present in only 10% of samples.

### Special stains

Perls’ Prussian blue staining in control livers showed rare intracellular hemosiderin, predominantly within flattened Kupffer cells (median 0.5 hemosiderin aggregates per sample; standard deviation/SD 2.6) (Plate 1F, inset). In infected dogs, hemosiderin was markedly increased, particularly associated with hypertrophic Kupffer cells (median 53 hemosiderin aggregates per sample; SD 35.2; *p* < 0.001; Figure 1F). Martius Scarlet Blue (MSB) staining revealed no fibrin thrombi in infected samples, and Gomori’s silver staining showed no disruption of the reticulin framework, including within centrilobular necrotic foci.

*Babesia* parasites were absent in controls and present in infected samples. In portal tracts and periportal zones, 70% of cases exhibited fewer than 10 intra-erythrocytic piroplasms per HPF (400x magnification), and 30% contained more than 10 red cell-associated parasites. In midzonal and centrilobular regions, 60% of samples exhibited few parasites, and 30% had many. The average parasite count was similar across hepatic zones (median 8.5 piroplasms per sample; SD 15.4).

### Immunohistochemistry overview

**Figure 2:**
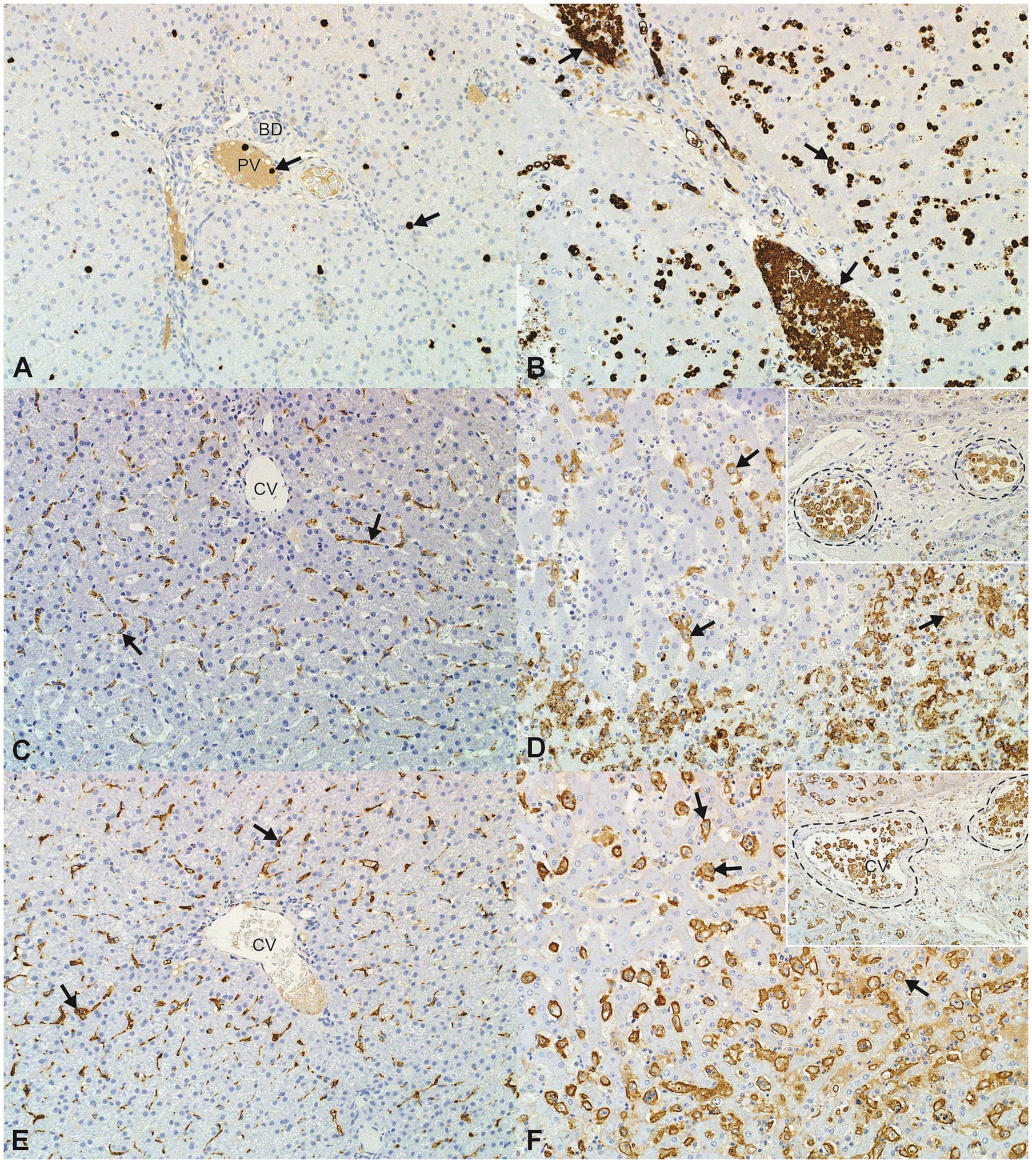
Immunohistochemical labelling of mononuclear phagocytes in the livers of *Babesia rossi*-infected dogs. Panels show representative immunolabelling patterns for MAC387, CD204, and Iba-1 in infected dogs (increased activated mononuclear phagocytes) compared with quiescent patterns in uninfected controls. BD, bile duct; CV, central vein; PV, portal vein. **(A)** (Control; portal tract and midzonal region). Rare MAC387-positive mononuclear cells in the PV and adjacent sinusoids (arrows). **(B)** (Infected; portal tract and midzonal region). Numerous MAC387-positive mononuclear cells in sinusoids and within the PV (arrows). **(C)** (Control; centrilobular and midzonal regions). Slender, quiescent CD204-reactive Kupffer cells lining the sinusoids (arrows). **(D)** (Infected; midzonal region). Increased numbers of CD204-reactive mononuclear phagocytes, including hypertrophic Kupffer cells, in sinusoids (arrows). Inset (infected; centrilobular region): large aggregates of CD204-positive monocytoid leukocytes within the CV (dashed circles). **(E)** (Control; centrilobular and midzonal regions). Slender, quiescent Iba-1-reactive Kupffer cells lining the sinusoids (arrows). **(F)** (Infected; midzonal region). Numerous plump, hypertrophic Iba-1-reactive mononuclear phagocytes in sinusoids (arrows). Inset (infected; centrilobular region): increased numbers of Iba-1-positive monocytoid leukocytes within the CV (dashed outlines).

Infected livers showed elevated median counts of immunoreactive mononuclear leukocytes compared to controls across all hepatic zones (Table 3). Increases included MAC387-positive cells (particularly portal; Figures 2A,B), CD3-positive T lymphocytes (especially midzonal), CD204- and Iba-1-positive monocyte–macrophages (mainly centrilobular and midzonal; Figures 2C–F), and CD20- and MUM1-positive B lymphocytes and plasma cells (especially centrilobular). Median counts for all markers exceeded the normal range established in control livers.

**Table 3:** Immunophenotypic quantification of leukocytes in control (*n* = 4) and *Babesia rossi*-infected (*n* = 10) liver samples.

Leukocyte marker		*Babesia rossi*-infected dogs	Controls		
	Hepatic zone	Median number of positive cells/HPF (25th-75th percentile)	Median number of positive cells/HPF (25th-75th percentile)	*P*-value* (Control vs. infected cases)	Magnitude of increase in infected cases
CD3	Portal tract	12 (7–15.5)	6 (3–10)	**0.042**	1.5-fold increase
	Midzonal	45.5 (20.5–60.3)	16.5 (13.25–20)	**<0.001**	2.8-fold increase
	Centrilobular	5.0 (3–12)	3 (0.25–9.5)	0.205	1.7-fold increase
CD20	Portal tract	4 (2.5–10)	1 (1–2.75)	**<0.001**	3.3-fold increase
	Midzonal	9.0 (5–11)	4.5 (2–5)	**0.001**	2.3-fold increase
	Centrilobular	11.5 (5–20.3)	1.5 (0–5.75)	**<0.001**	9.5-fold increase
CD204	Portal tract	10 (6–15.5)	7.5 (4–12.25)	0.158	1.3-fold increase
	Midzonal	125.5 (117.3–143.3)	69 (57–78)	**<0.001**	1.9-fold increase
	Centrilobular	14 (8–20.5)	4.5 (3–5.75)	**<0.001**	2.8-fold increase
Iba-1	Portal tract	11 (7–13)	9 (8–10)	0.251	1.3-fold increase
	Midzonal	142.4 (134–165)	107.5 (104.3–114.5)	**<0.001**	1.3-fold increase
	Centrilobular	12 (9–23)	6 (3–6)	**<0.001**	2.1-fold increase
MAC387	Portal tract	23 (12.5–49)	1 (1–2.8)	**<0.001**	26.5-fold increase
	Midzonal	192 (116–254.5)	22 (10.8–28.5)	**<0.001**	8.6-fold increase
	Centrilobular	16 (10.8–48)	1 (0.3–3.8)	**<0.001**	17-fold increase
MUM1	Portal tract	3.5 (1–7.5)	2 (1–3)	0.138	1.3-fold increase
	Midzonal	4 (3–5)	3 (2–6)	0.554	1.3-fold increase
	Centrilobular	4.5 (2–12)	1 (0–4.3)	**0.003**	3.5-fold increase

The median cell counts (per HPF/400x magnification) per hepatic zone (portal tract, midzonal and centrilobular areas), as well as the standard deviation, *P*-value, and magnitude of increase are listed. All values represent absolute cell counts.

HPF, high power field (400x magnification). ^*^Significant *P*-values (*P*-value <0.05) are in bold.

### Detailed results for individual leukocyte markers

#### MAC 387

In infected livers, MAC387-positive leukocytes (monocytes- macrophages, and rare neutrophils) ranged from 4.7–268.7 cells/HPF across all hepatic zones. Median counts were 26.5 cells in portal regions, 189 in midzonal areas, and 17 in centrilobular regions (Table 3). These cells were mainly intravascular with lesser perivascular involvement (Figure 2B). Overall, MAC387-positive cells increased 9.6-fold compared with controls (Table 4; Figures 2A,B).

**Table 4:** Immunophenotypic quantification of leukocytes in control (*n* = 4) and *Babesia rossi*-infected (*n* = 10) liver samples.

	*Babesia rossi*-infected cases	Controls		
	Median number of positive cells/HPF (25th-75th percentile)	Median number of positive cells/HPF (25th-75th percentile)	*P*-value* (Control vs. infected cases)	Magnitude of increase in infected cases
CD3	62.5 (41–85.5)	27.5 (18.5–38.8)	**<0.001**	2.4-fold increase
CD20	25 (17.8–46.5)	8 (5–13.3)	**<0.001**	4.5-fold increase
CD204	154 (140.8–178)	81.5 (67.8–94)	**<0.001**	1.9-fold increase
Iba-1	167 (152.8–198)	121 (114.5–129)	**<0.001**	1.4-fold increase
MAC387	264.5 (154.3–368.8)	24 (13.5–31)	**<0.001**	9.6-fold increase
MUM1	14.0 (7–34.8)	6.5 (3.25–14.3)	**0.005**	2-fold increase

The median cell counts (per HPF/400x magnification) in the liver as a whole (non-zonal), as well as the standard deviation, *P*-value, and magnitude of increase are listed. All values represent absolute cell counts.

HPF, high power field (400x magnification). ^*^Significant *P*-values (*P*-value <0.05) are in bold.

#### CD3

In infected livers, CD3-positive T-lymphocytes ranged from 1.7–111.7 immunoreactive cells/HPF, with median counts of 9 in portal regions, 47 in midzonal areas, and 5 in centrilobular regions. Only the midzonal median exceeded the control range (Table 3). These cells were mainly intravascular, with smaller numbers in the perivascular interstitium. Overall, T lymphocytes increased 2.4-fold relative to controls (Table 4).

#### CD204

In *Babesia*-infected livers, CD204-reactive monocyte–macrophages ranged from 3.3-177/HPF, with median counts of 10 in portal areas, 132.5 in midzonal regions, and 14 in centrilobular zones. Midzonal and centrilobular counts exceeded the control range (Table 3; Figures 2C,D). CD204-positive cells were increased predominantly within sinusoids, mainly representing hypertrophic Kupffer cells, and were occasionally observed in veins, but remained infrequent in the interstitium (Figure 2D). Overall, these cells showed a 1.9-fold increase compared with controls (Table 4).

#### Iba-1

Iba-1-reactive monocyte–macrophages and dendritic cells ranged from 4–180.3 cells/HPF, with median counts of 11.5 in portal tracts and centrilobular zones, and 145 midzonally. Centrilobular and midzonal counts exceeded the control range (Table 3 and Figures 2E,F). These cells, likely predominantly hypertrophic Kupffer cells, were concentrated in sinusoids, with smaller populations of non-Kupffer mononuclear leukocytes in perivascular and periductal areas and occasionally within veins (Figure 2F). Overall, Iba-1-positive cells increased 1.4-fold relative to controls.

#### CD20

In *Babesia*-infected samples, CD20-positive cells (which in our IHC laboratory include both B lymphocytes and plasma cells) ranged from 2–44.3 cells/HPF, with median counts of 5 in portal tracts, 9 in midzonal areas, and 9.5 in centrilobular areas, all higher than control values (Table 3). Immunoreactive cells were mainly perivascular and periductal, with only occasional intravascular labelling. Overall, CD20-positive cell numbers were increased 4.5-fold relative to controls (Table 4).

#### MUM1/IRF4

In *Babesia*-infected samples, MUM1-positive plasma cells (with rare immunoreactive differentiated B-lymphocytes) ranged from 0.3–86.3 cells/HPF, with median counts of 2.5, 4, and 3.5 cells/HPF in portal tracts, midzonal, and centrilobular areas, respectively. Only the centrilobular median approached the upper limit of control values (Table 3). Plasma cells were mainly perivascular and periductal, while lymphocyte-like MUM1-positive cells were more often intravascular. Overall numbers of MUM1-positive cells were approximately 2-fold higher than in the controls (Table 4).

## Discussion

The liver in fatal *B. rossi* infection exhibited a characteristic injury pattern reflecting both marked hemolytic anemia and significant immune activation, including expansion of mononuclear phagocytes and subtle increases in lymphocytes, highlighting engagement of both innate and adaptive immunity. The most prominent feature was a pronounced expansion of the mononuclear phagocyte system, with numerous enlarged Kupffer cells and infiltrating bone marrow–derived monocytes, consistent with similar monocyte–macrophage responses documented in the spleen, lung, bone marrow, and brain of dogs with fatal babesiosis (12–14, 38). Additional lesions, including hepatocellular and canalicular cholestasis, extramedullary erythropoiesis (EMH), widening of the spaces of Disse, and centrilobular hepatocyte necrosis or hydropic change, further define the hepatic response to systemic disease.

Immunophenotyping confirmed heterogeneity within hepatic monocyte/macrophage-lineage populations. MAC387-positive cells increased 9.6-fold compared to controls. Although MAC387 also labels neutrophils, these were only rarely identified in H&E-stained sections, indicating that the MAC387 counts predominantly represented monocyte–macrophages. In contrast, CD204- and Iba-1-positive cells increased more modestly, by 1.9- and 1.4-fold, respectively. Perl’s Prussian blue staining for ferric iron correlated strongly with these monocyte-macrophage populations. Since CD204 and Iba-1 preferentially label differentiated or maturing macrophages, the disproportionate rise in MAC387-positive cells suggests substantial recruitment or acute activation of newly arrived monocytes and monocyte-derived macrophages, consistent with MAC387 upregulation in circulating monocytes and inflammatory macrophages (40–43). This occurred despite the fact that peripheral monocytosis occurred in only 4 of the 10 cases. This may reflect additional MAC387-labelling of activated Kupffer cells or sequestered immature monocyte-lineage cells within hepatic sinusoids, which may have been mediated by endothelial activation (33). Notably, intravascular aggregates within the hepatic vasculature (including central and portal veins) were composed predominantly of monocytoid cells, as indicated by MAC387-, CD204-, and Iba-1-positive labelling, with fewer CD3-positive T cells and occasional CD20-positive B cells. These findings point to coordinated responses by resident and recruited myeloid cells during acute hemoprotozoal infection and suggest that intravascular leukostasis may contribute to local immune activation and microvascular changes. As has been shown in malaria and suggested in Babesia, leukocyte adhesion molecules are likely to play a role in this pathology (38, 44).

Lymphocyte populations were increased within infected livers. CD3-positive T cells were most prominent in midzonal sinusoids, whereas CD20-positive B lymphocytes (including plasma cells due to known marker cross-reactivity in our laboratory) and MUM-1-positive plasma cells accumulated predominantly in perivascular regions. The larger CD20-positive population relative to MUM1-positive cells indicates expansion of immature B lymphocytes, consistent with observations in the bone marrow and lungs of *B. rossi*–infected dogs (13, 14). Mechanistically, this aligns with established B-cell biology. B lymphocytes act as antigen-presenting cells and can differentiate into antibody-producing plasma cells that enhance phagocytosis. They also secrete cytokines early in infection, and reduced B-cell numbers are associated with decreased cytokine and IFN-dependent chemokine production and lower host survival (45). Activation occurs via direct antigen recognition or through macrophages, which initiate follicular B-cell responses (46), while protozoal DNA can stimulate B cells and induce macrophages to release IL-12, TNF-*α*, and NO (47), providing a plausible mechanism for local hepatic expansion, although systemic B-cell responses may have been impaired in these fatal cases. Activated macrophages, particularly hypertrophied Kupffer cells, likely amplify this response through antigen presentation, cytokine release, and chemokine-mediated lymphocyte recruitment. The prominence of intravascular CD3-positive T lymphocytes may reflect redistribution or sequestration, a mechanism proposed to explain reduced circulating CD3-positive lymphocytes in severe infection (48). In addition, there may be an expansion or activation of hepatic tissue-resident T cells, which possess high cytotoxic potential and contribute to defence against infections, providing further context for the overall accumulation of T cells within the liver despite declining peripheral counts (49).

Kupffer cell hypertrophy was a consistent feature, as reported in dogs (15), gerbils (24), and human malaria (50), and is also characteristic of sepsis (51). Activated Kupffer cells release TNF-α, IL-1, nitric oxide, reactive species, proteases, and lipid mediators, including prostaglandins and thromboxane (52). While these mediators contribute to early antimicrobial defense, excessive production, as in severe endotoxemia, can promote hepatocellular and endothelial injury (53). The abundant intracytoplasmic hemosiderin within Kupffer cells reflects accelerated erythrophagocytosis, documented across species including sheep (54), dogs (15), mice (55), and cattle (56), and together with EMH argues against iron-restricted erythropoiesis as the cause of non-regenerative anemia (14). The poorly regenerative anemia (unexpected in a hemolytic anemia) seen in *B. rossi* infections is thus likely to be due to dyserythropoiesis and/or iron trapping (14). Hepatocytic hemosiderin described in human malaria (57), but not in sepsis, further supports hemolysis as the primary driver of hepatic iron accumulation in hemoparasitic infections. Similar hepatic hemosiderin accumulation has been described in dogs that died due to *B. canis* infection (18).

Severe widening of the spaces of Disse, as observed in this study, has also been reported in bovine (56), gerbil (24), equine (27), and murine (55) babesiosis, as well as in human malaria (57) and sepsis (58). This dilation may result from increased capillary leakage, sinusoidal hypoperfusion with elevated intrasinusoidal pressure (58), and increased lymph production. Hypoalbuminemia, present in 90% of cases in this study, may reflect systemic protein loss due to increased vascular permeability and is further compounded by the fact that albumin is a negative acute phase protein, which decreases during inflammation; together these factors may exacerbate leakage by lowering capillary oncotic pressure (59). Consistent with this, 90% of infected cases also exhibited prominent lymphatic distension in portal tracts, likely reflecting increased drainage demands from fluid-filled spaces of Disse. Similar lymphatic changes have been reported in the livers of patients with endotoxemia and sepsis, which share pathophysiological parallels with complicated babesiosis, including a cytokine-driven inflammatory response that produces comparable clinical and histopathological findings across multiple organs (34). Endotoxins also impair lymphatic pumping and increase lymph production as part of the systemic inflammatory response (60).

Coagulative necrosis was prominent in centrilobular hepatocytes, and hydropic vacuolation was also most pronounced in this zone, consistent with hypoxia-associated injury. This distribution aligns with the severe hemolytic anemia in these dogs and may be exacerbated by reduced splanchnic and hepatic blood flow, as reported in human malaria (61), and by increased oxygen consumption within splanchnic tissues, as described in sepsis (62). Additional factors, including heightened metabolic demands, impaired oxygen offloading from hemoglobin, and decreased hepatic oxygen extraction during *B. rossi* infection (63), may further intensify local hypoxia. Mild increases in hepatocyte binucleation and mitotic figures were also observed; although these features only gain significance when quantitatively assessed in controlled experimental systems, they may indicate a compensatory increase in hepatocyte turnover in response to injury.

Hypoxic liver injury is reflected in elevated serum transaminases (AST, ALT) and LDH levels within 12–48 h post-injury (64). In our study, hepatic necrosis or hydropic degeneration were also associated with elevated transaminase concentrations. Similarly elevated liver enzymes, including ALP, ALT, and AST, have been reported in sepsis, even in the absence of hypoxemia, potentially due to a hypermetabolic state (65). Pro-inflammatory cytokines such as TNF-α and IL-6, released by activated Kupffer cells during sepsis, modulate hepatocyte function and contribute to dysregulation (65). In addition, endotoxins and pancreatic enzymes from an impaired gut barrier (due to hypoxia) may further damage already compromised hepatocytes and sinusoidal endothelial cells (66). While hepatic dysfunction rarely progresses to liver failure, it signals organ stress and may well contribute to the icterus commonly seen in babesiosis.

Bile accumulation was evident in all infected livers, most prominently within centrilobular hepatocytes (often accompanied by hemosiderin), within intercellular canaliculi, and, less commonly, in some portal tract bile ducts. This centrilobular cholestasis aligns with hypoxia-related impairment of ATP-dependent bile salt export (67) and parallels the cholestatic changes seen in sepsis, where endotoxins are thought to disrupt canalicular bile secretion, and promote ischemia (58). Similar hepatocellular bile pigmentation has been described in *B. canis* (18), sepsis (51), porcine (68) and equine babesiosis (27), and human malaria (32). All infected dogs were hyperbilirubinemic, and most were visibly icteric. Hyperbilirubinemia in *B. rossi* infection is associated with a poorer prognosis (34) and reflects both increased bilirubin production from hemolysis and reduced hepatic clearance, the latter likely associated with hepatocellular injury (69).

Nucleated red blood cells were present in all infected livers, appearing as individual intravascular cells, small intravascular aggregates, and occasional small extravascular clusters in all zones, findings consistent with circulating metarubricytes and mild hepatic EMH. Megakaryocytes were quite scarce. Hepatic EMH has been described in *B. canis* (15), murine babesiosis (55), human malaria (32), and other severe inflammatory states (70). The subtle EMH-like changes in *B. rossi* infection likely represent a mild reactive response to hypoxia and increased erythropoietic demand, occurring in the context of impaired marrow regeneration described in babesiosis and falciparum malaria (71), and potentially influenced by dyserythropoiesis and inflammatory mediators such as TNF-*α* and nitric oxide (5).

The study had several limitations. Only fatal cases were examined, preventing assessment of hepatic changes in sublethal or recovering infections. The control group was small and mismatched in age, sex, weight, and breed, introducing the possibility that some histological differences reflect baseline demographic or breed-related variation rather than infection-specific effects. In addition, because the study assesses tissues only at the time of death, it represents a single end-stage snapshot and therefore provides limited information about the earlier cellular events and mechanisms that drive disease evolution. The study did not describe the macroscopic changes of the liver at the time of post-mortem examination. Future studies using well-matched controls, experimental infections, and more detailed immunophenotyping and flow cytometry of hepatic myeloid and lymphoid populations would help distinguish changes associated with fatal disease from those occurring during effective immune resolution.

## Conclusion

The liver in *B. rossi*–infected dogs exhibits marked activation of tissue-resident Kupffer cells, with contributions from recruited or sequestered monocyte-macrophages and modest lymphocyte increases, reflecting coordinated innate and adaptive immune responses. Histopathological changes, including centrilobular necrosis, hepatocellular and canalicular cholestasis, and predominant hemosiderin accumulation in Kupffer cells, mirror lesions seen in severe sepsis, malaria, and other *Babesia* infections. Clinical and biochemical indicators, such as elevated hepatic enzymes, bilirubin, icterus, and hypoglycemia in ~25% of cases, confirm significant hepatic stress. Together with reported elevations in macrophage-associated cytokines (IL-10, IL-6, Monocyte Chemoattractant Protein-1), these findings indicate that liver injury in severe babesiosis results from hemolysis- and hypoxia-driven damage, exacerbated by activated Kupffer cells, recruited monocyte-macrophages, and lymphocytes. The liver is likely to be an organ that suffers bystander damage as a consequence of the host-driven response to infection, rather than be an organ whose dysfunction or failure directly results in death.

## Data Availability

The original contributions presented in the study are included in the article/Supplementary material, further inquiries can be directed to the corresponding author/s.
