# Supplementary material for: Liver histopathology in dogs with naturally acquired Babesia rossi infection
Source: Front Vet Sci. 2026 Jun 8;13:1765994. doi: 10.3389/fvets.2026.1765994 (PMC13283813; doi:10.3389/fvets.2026.1765994)
Supplement: Supplementary file 1 [file Table_1.DOCX]

##
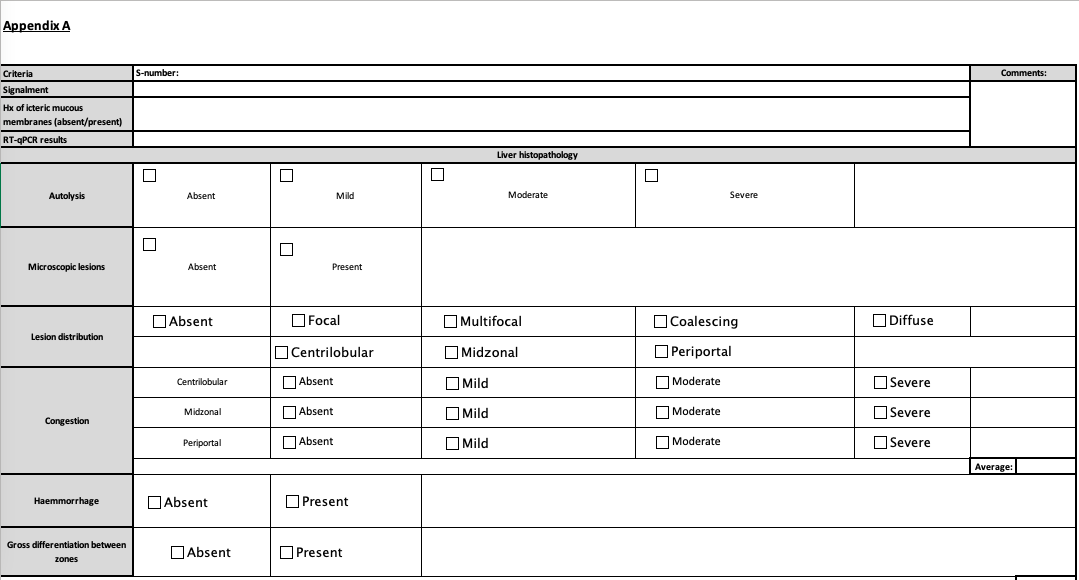

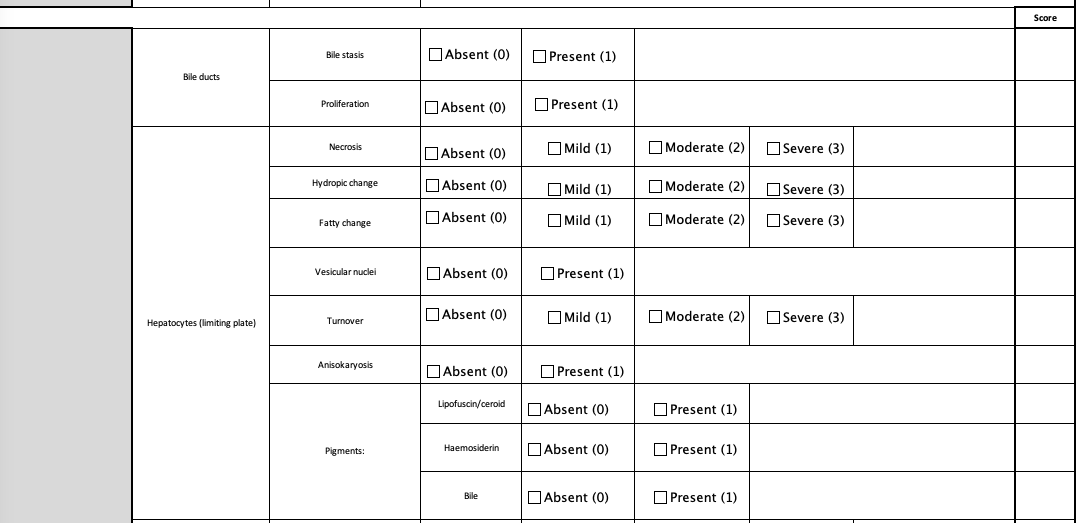

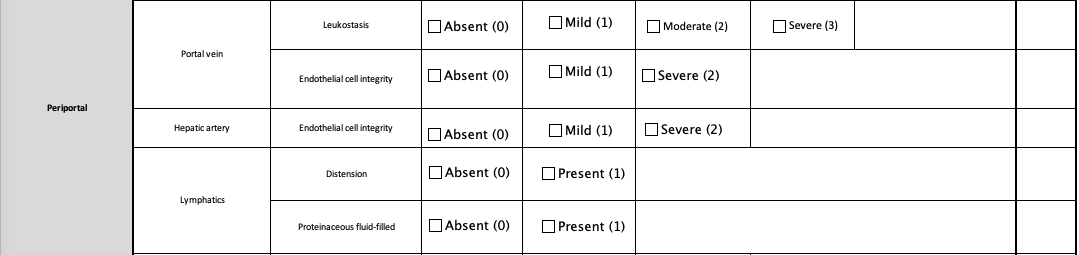
Supplemental Material 1 Histopathology score sheet


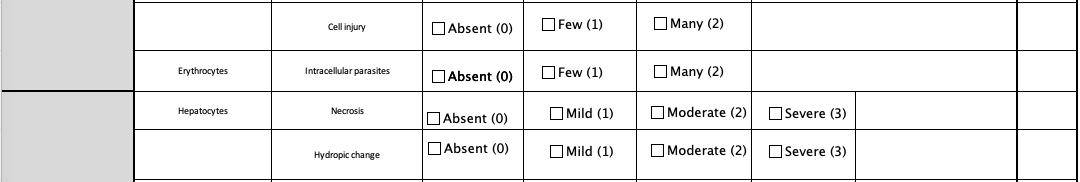

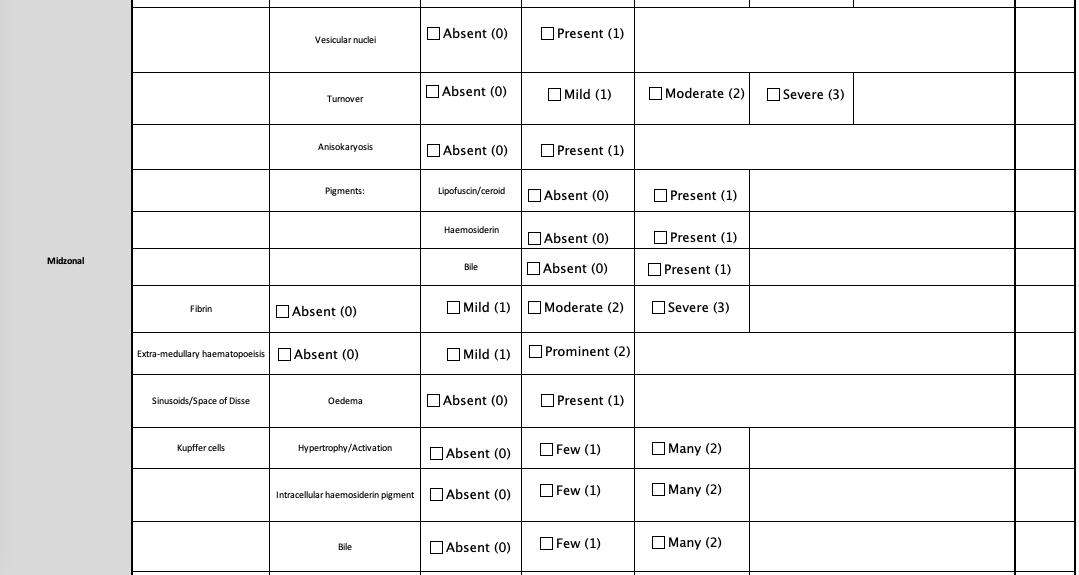

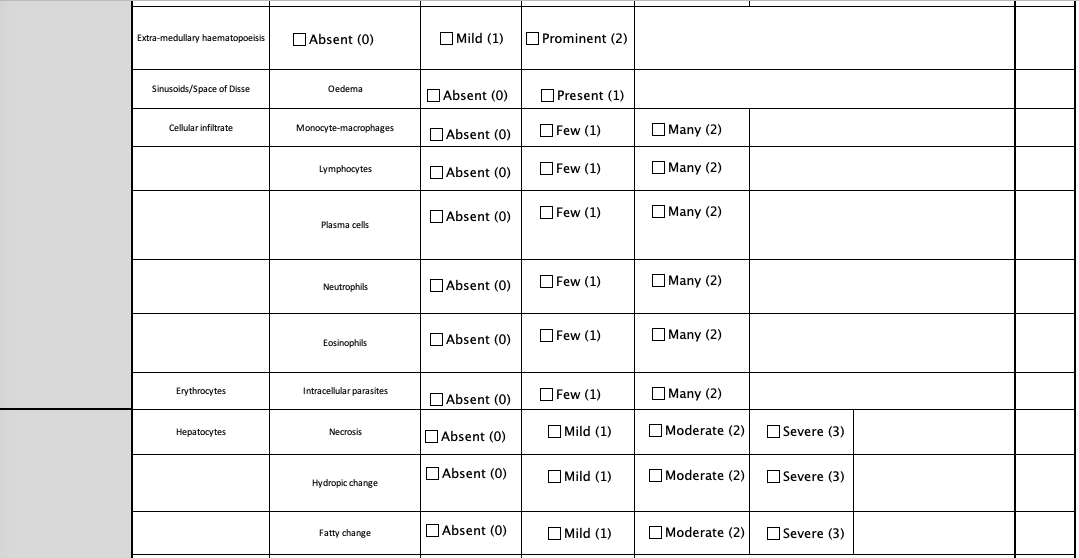


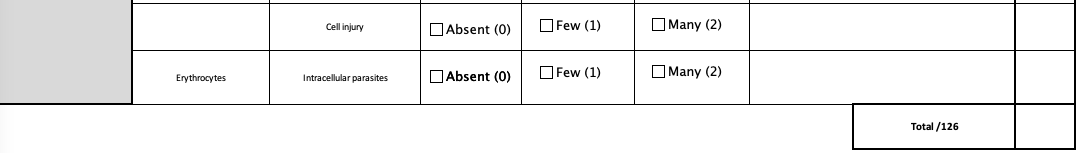

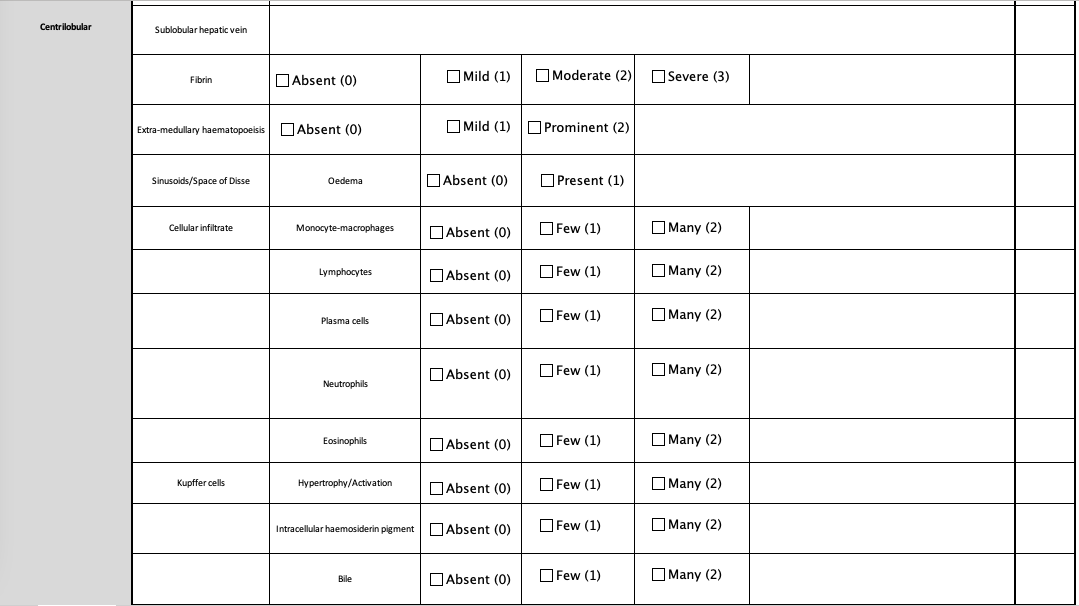

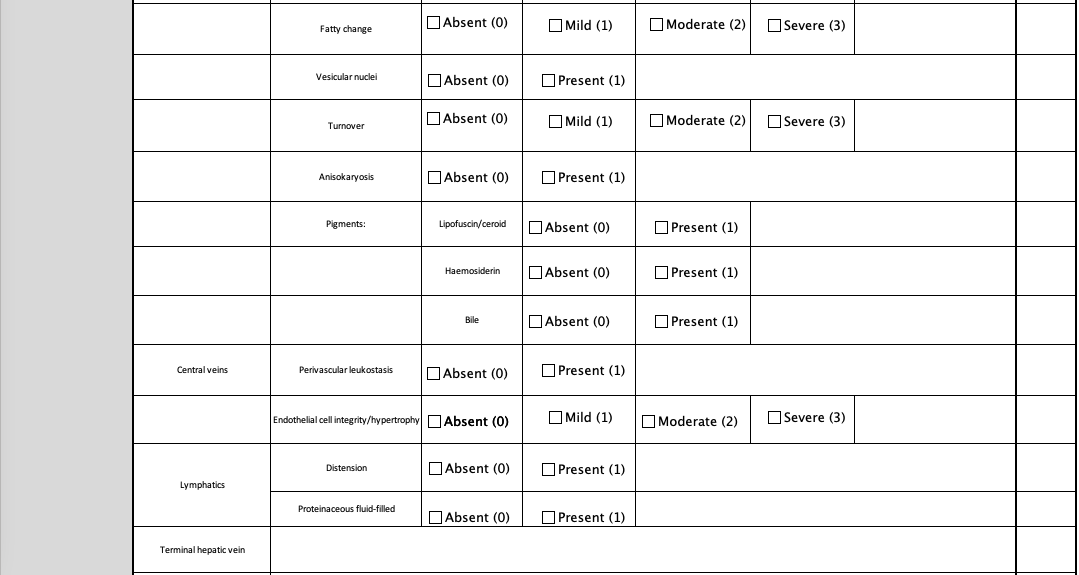


## Histopathology detailed scoring scheme

|  | | | | | |
| --- | --- | --- | --- | --- | --- |
| PARAMETER | GRADING/  SCORE | CRITERIA | | | |
| AUTOLYSIS |  |  | | | |
| Severity | Mild | Anatomical architecture easily distinguishable, cellular morphology well-defined. | | | |
|  | Moderate | Anatomical architecture still easily distinguishable, cellular morphology not well-defined, red blood cells still preserved, small numbers of putrefactive (Clostridial) bacteria may be present in some fields. | | | |
|  | Severe | Cellular morphology cannot be defined, anatomical architecture barely distinguishable, red blood cells lysed and difficult to identify, numerous putrefactive (Clostridial) bacteria present. | | | |
| HISTOPATHOLOGY | (All changes in comparison with normal control liver). | | | | |
| Microscopic lesions | Absent | No evidence of microscopic lesions when scanning on 40x and 100x magnification. | | | |
|  | Present | Evidence of microscopic lesions, regardless of severity or distribution. | | | |
| Lesion distribution (histologic)  *Will have lesion distribution too - periportal, centrilobular, midzonal* | Absent | No evidence of microscopic lesions. | | | |
|  | Focal | Single well circumscribed area affected. | | | |
|  | Multifocal | Multiple non-overlapping areas affected. | | | |
|  | Coalescing | Multiple overlapping areas affected. | | | |
|  | Diffuse | Entire section of tissue OR all specific anatomical sites affected | | | |
| Congestion  *Will have lesion distribution here too - periportal, centrilobular, midzonal*  *(Intravascular accumulation of rbcs)* | Absent | No evidence of congestion/hyperaemia | | | |
|  | Mild | Congestion present in <35% of blood vessels in all available histology sections | | | |
|  | Moderate | Congestion present in <70% of blood vessels in all available histology sections | | | |
|  | Severe | Congestion is present in most blood vessels (>70%) in all available histology sections.  **Note: check venules, arterioles and capillaries. If present in arterioles make a note (= hyperaemia).**  **Check sinusoids as well. If packed look at controls.**  **Need to look at density inside lumens** | | | |
| Haemorrhage = extravasated erythrocytes | Absent | No evidence of haemmorrhage | | | |
|  | Present | Evidence of erythrocytes present outside of the blood vessels | | | |
| Gross differentiation between the zones | Absent | Periportal, midzonal and centrilobular zones are not easily differentiable at first glance | | | |
|  | Present | Periportal, midzonal and centrilobular zones are easily differentiable and are noticeably different colours. | | | |
|  | | | | | |
| Periportal | | | | | |
| Bile ducts | Bile stasis | Absent (0) | | No evidence of bilirubin in the bile ducts | |
|  |  | Present (1) | | Evidence of bilirubin in the bile ducts | |
|  | Proliferation | Absent (0) | | No evidence of an increase in thickness of the bile duct walls | |
|  |  | Present (1) | | Evidence of an increase in the thickness of the bile duct walls or increase in number | |
| Hepatocytes (limiting plate)  (*40x magnification in 3 FOV)* | Necrosis  (*Eosinophilic, surrounding necrosis*) | Absent (0) | | No necrotic hepatocytes visualized | |
|  |  | Mild (1) | | Single cell necrosis | |
|  |  | Moderate (2) | | Necrosis of small clusters/groups of hepatocytes – 2-5 cells | |
|  |  | Severe (3) | | Necrosis of 6 or more cells | |
|  | Hydropic change  *(Feathery changes)* | Absent (0) | | No evidence of hydropic change present | |
|  |  | Mild (1) | | Hydropic changes present in 1 hepatocyte | |
|  |  | Moderate (2) | | Hydropic changes in small clusters/groups of hepatocytes – 2-5 cells | |
|  |  | Severe (3) | | Hydropic changes in and more than 6 hepatocytes | |
|  | Fatty change  *(Punched out holes)* | Absent (0) | | No evidence of fatty change present in any cells | |
|  |  | Mild (1) | | Fatty changes present in 1 hepatocyte | |
|  |  | Moderate (2) | | Fatty changes in small clusters/groups of hepatocytes – 2-5 cells | |
|  |  | Severe (3) | | Fatty changes in and more than 6 hepatocytes | |
|  | Vesicular nuclei  *(Euchromatic.*  *1.5x size of average of hepatocyte nucleus)* | Absent (0) | | No evidence of vesicular nuclei present (< and including 1 per FOV). | |
|  |  | Present (1) | | Evidence of vesicular nuclei present (>1 per FOV). | |
|  | Turnover  (*Mitotic bodies, binucleates*) | Absent (0) | | No evidence of any turnover present | |
|  |  | Mild (1) | | Evidence of turnover present in 1-2 hepatocytes per field | |
|  |  | Moderate (2) | | Evidence of turnover present in 2-5 hepatocytes per field | |
|  |  | Severe (3) | | Evidence of turnover present in more than 5 hepatocytes per field | |
|  | Anisokaryosis | Absent (0) | | No evidence of anisokaryosis present | |
|  |  | Present (1) | | Evidence of anisokaryosis | |
|  | Pigments: | | | | |
|  | Lipofuscin/ceroid | Absent (0) | | No evidence of pigment present | |
|  |  | Present (1) | | Evidence of pigment present | |
|  | Haemosiderin | Absent (0) | | No evidence of pigment present | |
|  |  | Present (1) | | Evidence of pigment present | |
|  | Bile canalicular | Absent (0) | | No evidence of bile present | |
|  |  | Present (1) | | Evidence of bile present | |
| Portal vein | Leukostasis  *Use similar sized vessels* | Absent (0) | | No evidence of leukostasis present. (Look out for normoblasts). | |
|  |  | Mild (1) | | Less than one quarter of the blood vessel’s lumen displays leukostasis at 40x magnification, when checking at least 3 FOV. | |
|  |  | Moderate (2) | | Half of the blood vessel’s lumen displays leukostasis at 40x magnification, when checking at least 3 FOV. | |
|  |  | Severe (3) | | More than and including three quarters of the blood vessel’s lumen displays leukostasis at 40x magnification, when checking at least 3 FOV. | |
|  |  | Cellular infiltrate | | Identify and describe predominant cellular component (mononuclear or PMN) in leukostatic blood vessels, based on HE (IMP will confirm later). | |
|  | Endothelial cell integrity/  hypertrophy  *(Endothelial hypertrophy = nucleus width is double or more than the normal thickness)* | Absent (0) | | No evidence of endothelial cell hypertrophy. Endothelial cell is diameter is less than RBC. | |
|  |  | Mild (1) | | Less than one third of endothelial cells display nuclear activation/hypertrophy. Endothelial cell diameter is the same as a RBC. | |
|  |  | Severe (2) | | More than two thirds of endothelial cells display nuclear activation/hypertrophy. Endothelial cell diameter is bigger than one RBC.  **Note: Comparing to control samples diameter of endothelial cells at central veins. Should be flat and the same diameter as a flat rbc.**  **Diameter <1 rbc = mild**  **Diameter >1 rbc = severe** | |
| Hepatic artery | Endothelial cell integrity/  hypertrophy  *(Endothelial hypertrophy = nucleus width is double or more than the normal thickness)* | Absent (0) | | No evidence of endothelial cell hypertrophy. | |
|  |  | Mild (1) | | Less than one third of endothelial cells display nuclear activation/hypertrophy. | |
|  |  | Moderate (2) | | One to two thirds of endothelial cells display nuclear activation/hypertrophy. | |
|  |  | Severe (3) | | More than two thirds of endothelial cells display nuclear activation/hypertrophy. | |
| Lymphatics  *(Subcapsular, portal and central venous areas. Will have* *endothelial cells)* | Distended | Absent (0) | | No evidence of lymphatic distension | |
|  |  | Present (1) | | Evidence of lymphatic distension | |
|  | Proteinaceous fluid-filled | Absent (0) | | No evidence of proteinaceous fluid present | |
|  |  | Present (1) | | Evidence of proteinaceous fluid present | |
| Fibrin  *(Confirmed on MSB histochemical stain)*  *Take note of where the fibrin is deposited* | Absent (0) | No evidence of fibrinous exudate | | | |
|  | Mild (1) | Affecting less than a third of the blood vessels (under 40x magnification, estimated over 3 x FOV). | | | |
|  | Moderate (2) | Affecting more than a third to two thirds of the blood vessels (under 40x magnification, estimated over 3 x FOV). | | | |
|  | Severe (3) | Affecting more than two thirds of the blood vessels (under 40x magnification, estimated over 3 x FOV). | | | |
| Extra-medullary haematopoeisis  *(Megakaryocytes and metarubricytes/normoblasts)* | Absent (0) | No evidence of extra-medullary haematopoeisis present intravascularly. | | |  |
|  | Mild (1) | Less than 5 megakaryocytes/metarubricytes/normoblasts seen in 3x FOV under 40x magnification | | |  |
|  | Prominent (2) | More than 5 megakaryocytes/metarubricytes/normoblasts seen in 3 x FOV under 40x magnification | | |  |
| Sinusoids/Spaces of Disse | Oedema  *(= amorphous clear to eosinophilic extravascular fluid)* | Absent (0) | No evidence of oedema | |  |
|  |  | Present (1) | Expansion of the perivascular areas by oedema fluid | |  |
| Cellular infiltrate  *(Confirmed with IHC)*  *40x magnification* | Monocyte-macrophages | Absent (0) | No monocytes/macrophages seen | | |
|  |  | Few (1) | Less than 20 monocytes/macrophages seen per FOV | | |
|  |  | Many (2) | More than and including 20 monocytes/macrophages seen per FOV | | |
|  | Lymphocytes | Absent (0) | No lymphocytes seen | | |
|  |  | Few (1) | Less than 20 lymphocytes seen per FOV | | |
|  |  | Many (2) | More than and including 20 lymphocytes seen per FOV | | |
|  | Plasma cells | Absent (0) | No plasma cells seen | | |
|  |  | Few (1) | Less than 20 plasma cells seen per FOV | | |
|  |  | Many (2) | More than and including 20 plasma cells seen per FOV | | |
|  | Neutrophils | Absent (0) | No neutrophils seen | | |
|  |  | Few (1) | Less than 20 neutrophils seen per FOV | | |
|  |  | Many (2) | More than and including 20 neutrophils seen per FOV | | |
|  | Eosinophils | Absent (0) | No eosinophils seen | | |
|  |  | Few (1) | 1-2 eosinophils seen per FOV | | |
|  |  | Many (2) | More than 2 eosinophils seen per FOV | | |
| Erythrocytes  *(Confirmed with LFB histochemical stain)*  *60x magnification* | Intraerythrocytic parasites | Absent (0) | No evidence of parasitized erythrocytes. | | |
|  |  | Few (1) | Less than 10 parasitized erythrocytes seen per FOV. | | |
|  |  | Many (2) | More than and including 10 parasitized erythrocytes seen per FOV. | | |
| Midzonal | | | | | |
| Hepatocytes  *40x magnification in 3x FOV* | Necrosis  (*Eosinophilic, surrounding necrosis*) | Absent (0) | No necrotic hepatocytes visualized | | |
|  |  | Mild (1) | Single cell necrosis | | |
|  |  | Moderate (2) | Necrosis of small clusters/groups of hepatocytes – 2-5 cells | | |
|  |  | Severe (3) | Necrosis of 6 or more cells | | |
|  | Hydropic change  *(Feathery changes)* | Absent (0) | No evidence of hydropic change present | | |
|  |  | Mild (1) | Hydropic changes present in 1 hepatocyte | | |
|  |  | Moderate (2) | Hydropic changes in small clusters/groups of hepatocytes – 2-5 cells | | |
|  |  | Severe (3) | Hydropic changes in and more than 6 hepatocytes | | |
|  | Fatty change  *(Punched out holes)* | Absent (0) | No evidence of fatty change present in any cells | | |
|  |  | Mild (1) | Fatty changes present in 1 hepatocyte | | |
|  |  | Moderate (2) | Fatty changes in small clusters/groups of hepatocytes – 2-5 cells | | |
|  |  | Severe (3) | Fatty changes in and more than 6 hepatocytes | | |
|  | Vesicular nuclei  *(Euchromatic.*  *1.5x size of average of hepatocyte nucleus)* | Absent (0) | No evidence of vesicular nuclei present (< and including 1 per FOV). | | |
|  |  | Present (1) | Evidence of vesicular nuclei present (>1 per FOV). | | |
|  | Turnover  (*Mitotic bodies, binucleates*) | Absent (0) | No evidence of any turnover present | | |
|  |  | Mild (1) | Evidence of turnover present in 1-2 hepatocytes per field | | |
|  |  | Moderate (2) | Evidence of turnover present in 2-5 hepatocytes per field | | |
|  |  | Severe (3) | Evidence of turnover present in more than 5 hepatocytes per field | | |
|  | Anisokaryosis | Absent (0) | No evidence of anisokaryosis present | | |
|  |  | Present (1) | Evidence of anisokaryosis | | |
|  | Pigments: |  |  | | |
|  | Lipofuscin/ceroid | Absent (0) | No evidence of pigment present | | |
|  |  | Present (1) | Evidence of pigment present | | |
|  | Haemosiderin | Absent (0) | No evidence of pigment present | | |
|  |  | Present (1) | Evidence of pigment present | | |
|  | Bile | Absent (0) | No evidence of bile present | | |
|  |  | Present (1) | Evidence of bile present | | |
| Fibrin  *(Confirmed on MSB histochemical stain)*  *Take note of where the fibrin is deposited* | Absent (0) | No evidence of fibrinous exudate | | | |
|  | Mild (1) | Affecting less than a third of the blood vessels (under 40x magnification, estimated over 3 x FOV). | | | |
|  | Moderate (2) | Affecting more than a third to two thirds of the blood vessels (under 40x magnification, estimated over 3 x FOV). | | | |
|  | Severe (3) | Affecting more than two thirds of the blood vessels (under 40x magnification, estimated over 3 x FOV). | | | |
| Extra-medullary haematopoeisis  *(Megakaryocytes and metarubricytes/normoblasts)* | Absent (0) | No evidence of extra-medullary haematopoeisis present intravascularly. | | |  |
|  | Mild (1) | Less than 5 megakaryocytes/metarubricytes/normoblasts seen in 3x FOV under 40x magnification | | |  |
|  | Prominent (2) | More than 5 megakaryocytes/metarubricytes/normoblasts seen in 3 x FOV under 40x magnification | | |  |
| Sinusoids/Spaces of Disse | Oedema  *(= amorphous clear to eosinophilic extravascular fluid)* | Absent (0) | No evidence of oedema | | |
|  |  | Present (1) | Expansion of the perivascular areas by oedema fluid | | |
| Kupffer cells | Hypertrophy/Activation  *(Look for pigment)* | Absent (0) | Less than one third of Kupffer cells display nuclear activation/hypertrophy. | | |
|  |  | Few (1) | One to two thirds of Kupffer cells display nuclear activation/hypertrophy. | | |
|  |  | Many (2) | More than two thirds of Kupffer cells display nuclear activation/hypertrophy. | | |
|  | Intracellular haemosiderin pigment | Absent (0) | No intracellular haemosiderin pigment visible | | |
|  |  | Few (1) | Intracellular haemosiderin pigment visible in less than 20% of cells | | |
|  |  | Many (2) | Intracellular haemosiderin pigment visible in more than 20% of cells | | |
|  | Bile | Absent (0) | No evidence of intracellular bile pigment | | |
|  |  | Few (1) | Intracellular bile pigment visible in less than 20% of cells | | |
|  |  | Many (2) | Intracellular bile pigment visible in more than 20% of cells | | |
|  | Cell injury *(necrosis/apoptosis)*  *Centrilobular bridging necrosis extending into midzonal* | Absent (0) | All cells intact and no evidence of injury | | |
|  |  | Few (1) | Evidence of cell injury in less than 20% of cells | | |
|  |  | Many (2) | Evidence of cell injury in more than 20% of cells | | |
| Erythrocytes  *(Confirmed with LFB histochemical stain)*  *60x magnification in 3x FOV* | Intraerythrocytic parasites | Absent (0) | No evidence of parasitized erythrocytes. | | |
|  |  | Few (1) | Less than 10 parasitized erythrocytes seen per FOV. | | |
|  |  | Many (2) | More than and including 10 parasitized erythrocytes seen per FOV. | | |
| Centrilobular | | | | | |
| Hepatocytes  *40x magnification in 3x FOV* | Necrosis  (*Eosinophilic, surrounding necrosis*) | Absent (0) | No necrotic hepatocytes visualized | | |
|  |  | Mild (1) | Single cell necrosis | | |
|  |  | Moderate (2) | Necrosis of small clusters/groups of hepatocytes – 2-5 cells | | |
|  |  | Severe (3) | Necrosis of 6 or more cells | | |
|  | Hydropic change  *(Feathery changes)* | Absent (0) | No evidence of hydropic change present | | |
|  |  | Mild (1) | Hydropic changes present in 1 hepatocyte | | |
|  |  | Moderate (2) | Hydropic changes in small clusters/groups of hepatocytes – 2-5 cells | | |
|  |  | Severe (3) | Hydropic changes in and more than 6 hepatocytes | | |
|  | Fatty change  *(Punched out holes)* | Absent (0) | No evidence of fatty change present in any cells | | |
|  |  | Mild (1) | Fatty changes present in 1 hepatocyte | | |
|  |  | Moderate (2) | Fatty changes in small clusters/groups of hepatocytes – 2-5 cells | | |
|  |  | Severe (3) | Fatty changes in and more than 6 hepatocytes | | |
|  | Vesicular nuclei  *(Euchromatic.*  *1.5x size of average of hepatocyte nucleus)* | Absent (0) | No evidence of vesicular nuclei present (< and including 1 per FOV). | | |
|  |  | Present (1) | Evidence of vesicular nuclei present (>1 per FOV). | | |
|  | Turnover  (*Mitotic bodies, binucleates*) | Absent (0) | No evidence of any turnover present | | |
|  |  | Mild (1) | Evidence of turnover present in 1-2 hepatocytes per field | | |
|  |  | Moderate (2) | Evidence of turnover present in 2-5 hepatocytes per field | | |
|  |  | Severe (3) | Evidence of turnover present in more than 5 hepatocytes per field | | |
|  | Anisokaryosis | Absent (0) | No evidence of anisokaryosis present | | |
|  |  | Present (1) | Evidence of anisokaryosis | | |
|  | Pigments: |  |  | | |
|  | Lipofuscin/ceroid | Absent (0) | No evidence of pigment present | | |
|  |  | Present (1) | Evidence of pigment present | | |
|  | Haemosiderin | Absent (0) | No evidence of pigment present | | |
|  |  | Present (1) | Evidence of pigment present | | |
|  | Bile | Absent (0) | No evidence of bile present | | |
|  |  | Present (1) | Evidence of bile present | | |
| Central vein | Endothelial cell integrity/  hypertrophy  *Endothelial hypertrophy = nucleus width is double or more than the normal thickness.* | Absent (0) | No evidence of endothelial cell hypertrophy. | | |
|  |  | Mild (1) | Less than one third of endothelial cells display nuclear activation/hypertrophy. | | |
|  |  | Moderate (2) | One to two thirds of endothelial cells display nuclear activation/hypertrophy. | | |
|  |  | Severe (3) | More than two thirds of endothelial cells display nuclear activation/hypertrophy. | | |
|  | Leukostasis  *Use similar sized vessels* | Absent (0) | No evidence of leukostasis present. (Look out for normoblasts). | | |
|  |  | Mild (1) | Less than one quarter of the blood vessel’s lumen displays leukostasis at 40x magnification, when checking at least 3 FOV. | | |
|  |  | Moderate (2) | Half of the blood vessel’s lumen displays leukostasis at 40x magnification, when checking at least 3 FOV. | | |
|  |  | Severe (3) | More than and including three quarters of the blood vessel’s lumen displays leukostasis at 40x magnification, when checking at least 3 FOV. | | |
|  |  | Cellular infiltrate | Identify and describe predominant cellular component (mononuclear or PMN) in leukostatic blood vessels, based on HE (IMP will confirm later). | | |
| Lymphatics  *(Subcapsular, portal and central venous areas. Will have* *endothelial cells)* | Distended | Absent (0) | No evidence of lymphatic distension | | |
|  |  | Present (1) | Evidence of lymphatic distension | | |
|  | Proteinaceous fluid-filled | Absent (0) | No evidence of proteinaceous fluid present | | |
|  |  | Present (1) | Evidence of proteinaceous fluid present | | |
| Terminal hepatic vein |  |  |  | | |
| Sublobular hepatic vein |  |  |  | | |
| Fibrin  *(Confirmed on MSB histochemical stain)*  *Take note of where the fibrin is deposited* | Absent (0) | No evidence of fibrinous exudate | | | |
|  | Mild (1) | Affecting less than a third of the blood vessels (under 40x magnification, estimated over 3 x FOV). | | | |
|  | Moderate (2) | Affecting more than a third to two thirds of the blood vessels (under 40x magnification, estimated over 3 x FOV). | | | |
|  | Severe (3) | Affecting more than two thirds of the blood vessels (under 40x magnification, estimated over 3 x FOV). | | | |
| Extra-medullary haematopoeisis  *(Megakaryocytes and metarubricytes/normoblasts)* | Absent (0) | No evidence of extra-medullary haematopoeisis present intravascularly. | | | |
|  | Mild (1) | Less than 5 megakaryocytes/metarubricytes/normoblasts seen in 3x FOV under 40x magnification | | | |
|  | Prominent (2) | More than 5 megakaryocytes/metarubricytes/normoblasts seen in 3 x FOV under 40x magnification | | | |
| Sinusoids/Spaces of Disse | Oedema  *(= amorphous clear to eosinophilic extravascular fluid)* | Absent (0) | No evidence of oedema | | |
|  |  | Present (1) | Expansion of the perivascular areas by oedema fluid | | |
| Cellular infiltrate  *(Confirmed with IHC)*  *40x magnification* | Monocyte-macrophages | Absent (0) | No monocytes/macrophages seen | | |
|  |  | Few (1) | Less than 20 monocytes/macrophages seen per FOV | | |
|  |  | Many (2) | More than and including 20 monocytes/macrophages seen per FOV | | |
|  | Lymphocytes | Absent (0) | No lymphocytes seen | | |
|  |  | Few (1) | Less than 20 lymphocytes seen per FOV | | |
|  |  | Many (2) | More than and including 20 lymphocytes seen per FOV | | |
|  | Plasma cells | Absent (0) | No plasma cells seen | | |
|  |  | Few (1) | Less than 20 plasma cells seen per FOV | | |
|  |  | Many (2) | More than and including 20 plasma cells seen per FOV | | |
|  | Neutrophils | Absent (0) | No neutrophils seen | | |
|  |  | Few (1) | Less than 20 neutrophils seen per FOV | | |
|  |  | Many (2) | More than and including 20 neutrophils seen per FOV | | |
|  | Eosinophils | Absent (0) | No eosinophils seen | | |
|  |  | Few (1) | 1-2 eosinophils seen per FOV | | |
|  |  | Many (2) | More than 2 eosinophils seen per FOV | | |
| Kupffer cells | Hypertrophy/Activation  *(Look for pigment)* | Absent (0) | Less than one third of Kupffer cells display nuclear activation/hypertrophy. | | |
|  |  | Few (1) | One to two thirds of Kupffer cells display nuclear activation/hypertrophy. | | |
|  |  | Many (2) | More than two thirds of Kupffer cells display nuclear activation/hypertrophy. | | |
|  | Intracellular haemosiderin pigment | Absent (0) | No intracellular haemosiderin pigment visible | | |
|  |  | Few (1) | Intracellular haemosiderin pigment visible in less than 20% of cells | | |
|  |  | Many (2) | Intracellular haemosiderin pigment visible in more than 20% of cells | | |
|  | Bile | Absent (0) | No evidence of intracellular bile pigment | | |
|  |  | Few (1) | Intracellular bile pigment visible in less than 20% of cells | | |
|  |  | Many (2) | Intracellular bile pigment visible in more than 20% of cells | | |
|  | Cell injury *(necrosis/apoptosis)*  *Centrilobular bridging necrosis extending into midzonal* | Absent (0) | All cells intact and no evidence of injury | | |
|  |  | Few (1) | Evidence of cell injury in less than 20% of cells | | |
|  |  | Many (2) | Evidence of cell injury in more than 20% of cells | | |
| Erythrocytes  *(Confirmed with LFB histochemical stain)*  *60x magnification in 3x FOV* | Intraerythrocytic parasites | Absent (0) | No evidence of parasitized erythrocytes. | | |
|  |  | Few (1) | Less than 10 parasitized erythrocytes seen per FOV. | | |
|  |  | Many (2) | More than and including 10 parasitized erythrocytes seen per FOV. | | |
