# Supplementary material for: Liver histopathology in dogs with naturally acquired Babesia rossi infection
Source: Front Vet Sci. 2026 Jun 8;13:1765994. doi: 10.3389/fvets.2026.1765994 (PMC13283813; doi:10.3389/fvets.2026.1765994)
Supplement: Supplementary file 2 [file Table_2.DOCX]

### Supplimentary Data 2 – Immunohistochemistry results by case

### CD3

###### *Quantification of CD3-immunoreactive T-lymphocytes in the* B. rossi*-infected livers (n=10) compared to the controls (n=4). The median number of positive cells (per HPF/400x magnification) per hepatic zone (portal tract, midzonal and centrilobular), as well as the* P*-values are listed. All values represent absolute cell counts.*

| ***B. rossi*-infected cases** | | | **Control cases** |  |
| --- | --- | --- | --- | --- |
| **Infected dogs**  **(Case no.)** | **Hepatic zone** | **Median number of positive cells/HPF** | **Median number of positive cells/HPF** | ***P*-value* comparing the control vs. infected cases** |
| Case 20 | Portal tract | 2.5 | 6.0 | **0.042** |
|  | Midzonal | 12.3 | 16.5 | **<0.001** |
|  | Centrilobular | 7.0 | 3.0 | 0.205 |
| Case 50 | Portal tract | 8.3 | 6.0 | **0.042** |
|  | Midzonal | 14.7 | 16.5 | **<0.001** |
|  | Centrilobular | 8.3 | 3.0 | 0.205 |
| Case 54 | Portal tract | 23.7 | 6.0 | **0.042** |
|  | Midzonal | 111.7 | 16.5 | **<0.001** |
|  | Centrilobular | 5.7 | 3.0 | 0.205 |
| Case 59 | Portal tract | 10.0 | 6.0 | **0.042** |
|  | Midzonal | 23.0 | 16.5 | **<0.001** |
|  | Centrilobular | 13.3 | 3.0 | 0.205 |
| Case 61 | Portal tract | 21.3 | 6.0 | **0.042** |
|  | Midzonal | 55.7 | 16.5 | **<0.001** |
|  | Centrilobular | 4.0 | 3.0 | 0.205 |
| Case 88 | Portal tract | 12.0 | 6.0 | **0.042** |
|  | Midzonal | 36.3 | 16.5 | **<0.001** |
|  | Centrilobular | 6.7 | 3.0 | 0.205 |
| Case 91 | Portal tract | 9.3 | 6.0 | **0.042** |
|  | Midzonal | 29.3 | 16.5 | **<0.001** |
|  | Centrilobular | 7.0 | 3.0 | 0.205 |
| Case 106 | Portal tract | 14.5 | 6.0 | **0.042** |
|  | Midzonal | 61.3 | 16.5 | **<0.001** |
|  | Centrilobular | 10.7 | 3.0 | 0.205 |
| Case 110 | Portal tract | 9.0 | 6.0 | **0.042** |
|  | Midzonal | 65.3 | 16.5 | **<0.001** |
|  | Centrilobular | 9.0 | 3.0 | 0.205 |
| Case 117 | Portal tract | 12.3 | 6.0 | **0.042** |
|  | Midzonal | 54.3 | 16.5 | **<0.001** |
|  | Centrilobular | 1.7 | 3.0 | 0.205 |
| Total medians | Portal tract | 9 | 6.0 | **0.042** |
|  | Midzonal | 47 | 16.5 | **<0.001** |
|  | Centrilobular | 5 | 3.0 | 0.205 |

^Abbreviations: HPF = high power field (400x magnification).^

^*Significant^ *^P^*^-values (^*^P^*^-value <0.05) are in bold.^

### CD20

Quantification of CD20-immunoreactive B-lymphocytes and plasma cells in the B. rossi-infected livers (n=10) compared to the controls (n=4). The median number of positive cells (per HPF/400x magnification) per hepatic zone (portal tract, midzonal and centrilobular), as well as the P-values are listed. All values represent absolute cell counts.

| ***B. rossi*-infected cases** | | | **Control cases** |  |
| --- | --- | --- | --- | --- |
| **Infected dogs**  **(Case no.)** | **Hepatic zone** | **Median number of positive cells/HPF** | **Median number of positive cells/HPF** | ***P*-value* comparing the control vs. infected cases** |
| Case 20 | Portal tract | 9.3 | 1.5 | **<0.001** |
|  | Midzonal | 5.7 | 4.0 | **0.001** |
|  | Centrilobular | 35.3 | 1.0 | **<0.001** |
| Case 50 | Portal tract | 3.7 | 1.5 | **<0.001** |
|  | Midzonal | 3.0 | 4.0 | **0.001** |
|  | Centrilobular | 6.0 | 1.0 | **<0.001** |
| Case 54 | Portal tract | 8.3 | 1.5 | **<0.001** |
|  | Midzonal | 14.3 | 4.0 | **0.001** |
|  | Centrilobular | 12.0 | 1.0 | **<0.001** |
| Case 59 | Portal tract | 2.3 | 1.5 | **<0.001** |
|  | Midzonal | 3.0 | 4.0 | **0.001** |
|  | Centrilobular | 16.0 | 1.0 | **<0.001** |
| Case 61 | Portal tract | 2.0 | 1.5 | **<0.001** |
|  | Midzonal | 9.3 | 4.0 | **0.001** |
|  | Centrilobular | 8.3 | 1.0 | **<0.001** |
| Case 88 | Portal tract | 3.3 | 1.5 | **<0.001** |
|  | Midzonal | 9.3 | 4.0 | **0.001** |
|  | Centrilobular | 3.7 | 1.0 | **<0.001** |
| Case 91 | Portal tract | 4.7 | 1.5 | **<0.001** |
|  | Midzonal | 9.3 | 4.0 | **0.001** |
|  | Centrilobular | 14.0 | 1.0 | **<0.001** |
| Case 106 | Portal tract | 4.7 | 1.5 | **<0.001** |
|  | Midzonal | 10.3 | 4.0 | **0.001** |
|  | Centrilobular | 6.7 | 1.0 | **<0.001** |
| Case 110 | Portal tract | 24.0 | 1.5 | **<0.001** |
|  | Midzonal | 18.3 | 4.0 | **0.001** |
|  | Centrilobular | 44.3 | 1.0 | **<0.001** |
| Case 117 | Portal tract | 41.0 | 1.5 | **<0.001** |
|  | Midzonal | 8.0 | 4.0 | **0.001** |
|  | Centrilobular | 11.7 | 1.0 | **<0.001** |
| Total medians | Portal tract | 5.0 | 1.5 | **<0.001** |
|  | Midzonal | 9.0 | 4.0 | **0.001** |
|  | Centrilobular | 9.5 | 1.0 | **<0.001** |

^Abbreviations: HPF = high power field (400x magnification); *Significant^ *^P^*^-values (^*^P^*^-value <0.05) are in bold.^

### CD204

| ***B. rossi*-infected cases** | | | **Control cases** |  |
| --- | --- | --- | --- | --- |
| **Infected dogs (Case no.)** | **Hepatic zone** | **Median number of positive cells/HPF** | **Median number of positive cells/HPF** | ***P*-value* comparing the control vs. infected cases** |
| Case 20 | Portal tract | 8.0 | 7.5 | 0.158 |
|  | Midzonal | 88.0 | 71.0 | **<0.001** |
|  | Centrilobular | 13.0 | 5.0 | **<0.001** |
| Case 50 | Portal tract | 4.0 | 7.5 | 0.158 |
|  | Midzonal | 77.3 | 71.0 | **<0.001** |
|  | Centrilobular | 3.3 | 5.0 | **<0.001** |
| Case 54 | Portal tract | 4.3 | 7.5 | 0.158 |
|  | Midzonal | 131.0 | 71.0 | **<0.001** |
|  | Centrilobular | 10.3 | 5.0 | **<0.001** |
| Case 59 | Portal tract | 15.0 | 7.5 | 0.158 |
|  | Midzonal | 128.7 | 71.0 | **<0.001** |
|  | Centrilobular | 16.0 | 5.0 | **<0.001** |
| Case 61 | Portal tract | 25.5 | 7.5 | 0.158 |
|  | Midzonal | 128.3 | 71.0 | **<0.001** |
|  | Centrilobular | 49.0 | 5.0 | **<0.001** |
| Case 88 | Portal tract | 14.3 | 7.5 | 0.158 |
|  | Midzonal | 126.0 | 71.0 | **<0.001** |
|  | Centrilobular | 15.7 | 5.0 | **<0.001** |
| Case 91 | Portal tract | 11.0 | 7.5 | 0.158 |
|  | Midzonal | 140.3 | 71.0 | **<0.001** |
|  | Centrilobular | 16.3 | 5.0 | **<0.001** |
| Case 106 | Portal tract | 10.0 | 7.5 | 0.158 |
|  | Midzonal | 130.3 | 71.0 | **<0.001** |
|  | Centrilobular | 7.7 | 5.0 | **<0.001** |
| Case 110 | Portal tract | 11.7 | 7.5 | 0.158 |
|  | Midzonal | 132.7 | 71.0 | **<0.001** |
|  | Centrilobular | 12.7 | 5.0 | **<0.001** |
| Case 117 | Portal tract | 11.3 | 7.5 | 0.158 |
|  | Midzonal | 177.0 | 71.0 | **<0.001** |
|  | Centrilobular | 23.0 | 5.0 | **<0.001** |
| Total medians | Portal tract | 10 | 7.5 | 0.158 |
|  | Midzonal | 132.5 | 71.0 | **<0.001** |
|  | Centrilobular | 14 | 5.0 | **<0.001** |

###### *Quantification of CD204-immunoreactive macrophages in the* B. rossi*-infected livers (n=10) compared to the controls (n=4). The median number of positive cells (per HPF/400x magnification) per hepatic zone (portal tract, midzonal and centrilobular), as well as the* P*-values are listed. All values represent absolute cell counts.*

^Abbreviations: HPF = high power field (400x magnification)^**^.^**

^*Significant^ *^P^*^-values (^*^P^*^-value <0.05) are in bold.^

### Iba-1

###### *Quantification of Iba-1-immunoreactive monocyte-macrophages and dendritic cells in the* B. rossi*-infected livers (n=10) compared to the controls (n=4). The median number of positive cells (per HPF/400x magnification) per hepatic zone (portal tract, midzonal and centrilobular), as well as the* P*-values are listed. All values represent absolute cell counts.*

| ***B. rossi-*infected cases** | | | **Control cases** |  |
| --- | --- | --- | --- | --- |
| **Infected dogs**  **(Case no.)** | **Hepatic zone** | **Median number of positive cells/HPF** | **Median number of positive cells/HPF** | ***P*-value* comparing the control vs. infected cases** |
| Case 20 | Portal tract | 7.5 | 9.0 | 0.251 |
|  | Midzonal | 137.0 | 107.5 | **<0.001** |
|  | Centrilobular | 7.3 | 5.5 | **<0.001** |
| Case 50 | Portal tract | 6.3 | 9.0 | 0.251 |
|  | Midzonal | 132.7 | 107.5 | **<0.001** |
|  | Centrilobular | 8.5 | 5.5 | **<0.001** |
| Case 54 | Portal tract | 9.7 | 9.0 | 0.251 |
|  | Midzonal | 180.3 | 107.5 | **<0.001** |
|  | Centrilobular | 5.0 | 5.5 | **<0.001** |
| Case 59 | Portal tract | 14.0 | 9.0 | 0.251 |
|  | Midzonal | 139.3 | 107.5 | **<0.001** |
|  | Centrilobular | 24.7 | 5.5 | **<0.001** |
| Case 61 | Portal tract | 13.0 | 9.0 | 0.251 |
|  | Midzonal | 154.0 | 107.5 | **<0.001** |
|  | Centrilobular | 49.0 | 5.5 | **<0.001** |
| Case 88 | Portal tract | 13.3 | 9.0 | 0.251 |
|  | Midzonal | 169.3 | 107.5 | **<0.001** |
|  | Centrilobular | 13.3 | 5.5 | **<0.001** |
| Case 91 | Portal tract | 10.3 | 9.0 | 0.251 |
|  | Midzonal | 157.3 | 107.5 | **<0.001** |
|  | Centrilobular | 21.5 | 5.5 | **<0.001** |
| Case 106 | Portal tract | 14.0 | 9.0 | 0.251 |
|  | Midzonal | 139.3 | 107.5 | **<0.001** |
|  | Centrilobular | 10.0 | 5.5 | **<0.001** |
| Case 110 | Portal tract | 4.0 | 9.0 | 0.251 |
|  | Midzonal | 119.0 | 107.5 | **<0.001** |
|  | Centrilobular | 14.3 | 5.5 | **<0.001** |
| Case 117 | Portal tract | 11.7 | 9.0 | 0.251 |
|  | Midzonal | 151.0 | 107.5 | **<0.001** |
|  | Centrilobular | 26.3 | 5.5 | **<0.001** |
| Total medians | Portal tract | 11.5 | 9.0 | 0.251 |
|  | Midzonal | 145.0 | 107.5 | **<0.001** |
|  | Centrilobular | 11.5 | 5.5 | **<0.001** |

^Abbreviations: HPF = high power field (400x magnification).^

^*Significant^ *^P^*^-values (^*^P^*^-value <0.05) are in bold.^

### MAC387

###### *Quantification of MAC387-immunoreactive leucocytes (predominantly monocyte-macrophages**) in the* B. rossi*-infected livers (n=10) compared to the controls (n=4). The median number of positive cells (per HPF/400x magnification) per hepatic zone (portal tract, midzonal and centrilobular), as well as the* P*-values are listed. All values represent absolute cell counts.*

| ***B. rossi*-infected cases** | | | **Control cases** |  |
| --- | --- | --- | --- | --- |
| **Infected dogs**  **(Case no.)** | **Hepatic zone** | **Median number of positive cells/HPF** | **Median number of positive cells/HPF** | ***P*-value* comparing the control vs. infected cases** |
| Case 20 | Portal tract | 4.7 | 1.0 | **<0.001** |
|  | Midzonal | 87.7 | 22.0 | **<0.001** |
|  | Centrilobular | 10.3 | 1.0 | **<0.001** |
| Case 50 | Portal tract | 91.7 | 1.0 | **<0.001** |
|  | Midzonal | 268.7 | 22.0 | **<0.001** |
|  | Centrilobular | 66.0 | 1.0 | **<0.001** |
| Case 54 | Portal tract | 35.0 | 1.0 | **<0.001** |
|  | Midzonal | 205.3 | 22.0 | **<0.001** |
|  | Centrilobular | 14.3 | 1.0 | **<0.001** |
| Case 59 | Portal tract | 65.0 | 1.0 | **<0.001** |
|  | Midzonal | 275.7 | 22.0 | **<0.001** |
|  | Centrilobular | 118.3 | 1.0 | **<0.001** |
| Case 61 | Portal tract | 36.7 | 1.0 | **<0.001** |
|  | Midzonal | 282.7 | 22.0 | **<0.001** |
|  | Centrilobular | 50.3 | 1.0 | **<0.001** |
| Case 88 | Portal tract | 12.3 | 1.0 | **<0.001** |
|  | Midzonal | 152.7 | 22.0 | **<0.001** |
|  | Centrilobular | 13.3 | 1.0 | **<0.001** |
| Case 91 | Portal tract | 26.7 | 1.0 | **<0.001** |
|  | Midzonal | 253.3 | 22.0 | **<0.001** |
|  | Centrilobular | 9.7 | 1.0 | **<0.001** |
| Case 106 | Portal tract | 34.7 | 1.0 | **<0.001** |
|  | Midzonal | 114 | 22.0 | **<0.001** |
|  | Centrilobular | 15.7 | 1.0 | **<0.001** |
| Case 110 | Portal tract | 11.7 | 1.0 | **<0.001** |
|  | Midzonal | 101.3 | 22.0 | **<0.001** |
|  | Centrilobular | 16.0 | 1.0 | **<0.001** |
| Case 117 | Portal tract | 116.3 | 1.0 | **<0.001** |
|  | Midzonal | 193.0 | 22.0 | **<0.001** |
|  | Centrilobular | 38.7 | 1.0 | **<0.001** |
| Total medians | Portal tract | 26.5 | 1.0 | **<0.001** |
|  | Midzonal | 189.0 | 22.0 | **<0.001** |
|  | Centrilobular | 17.0 | 1.0 | **<0.001** |

^Abbreviations: HPF = high power field (400x magnification).^

^*Significant^ *^P^*^-values (^*^P^*^-value <0.05) are in bold.^

^** Generally, apart from 2/10 cases, few neutrophils were observed in the HE sections, so the MAC387 immunoreactivity refers largely to mononuclear phagocytes (monocyte-macrophages).^

### MUM-1

###### *Quantification of MUM-1-immunoreactive plasma cells and mature B-lymphocytes in the* B. rossi*-infected livers (n=10) compared to the controls (n=4). The median number of positive cells (per HPF/400x magnification) per hepatic zone (portal tract, midzonal and centrilobular), as well as the* P*-values are listed. All values represent absolute cell counts.*

| ***B. rossi*-infected cases** | | | **Control cases** |  |
| --- | --- | --- | --- | --- |
| **Infected dogs**  **(Case no.)** | **Hepatic zone** | **Median number of positive cells/HPF** | **Median number of positive cells/HPF** | ***P*-value* comparing the control vs. infected cases** |
| Case 20 | Portal tract | 5.0 | 2 | 0.138 |
|  | Midzonal | 4.7 | 3 | 0.554 |
|  | Centrilobular | 28.7 | 1 | **0.003** |
| Case 50 | Portal tract | 5.7 | 2 | 0.138 |
|  | Midzonal | 3.3 | 3 | 0.554 |
|  | Centrilobular | 8.3 | 1 | **0.003** |
| Case 54 | Portal tract | 0.3 | 2 | 0.138 |
|  | Midzonal | 5.7 | 3 | 0.554 |
|  | Centrilobular | 1.7 | 1 | **0.003** |
| Case 59 | Portal tract | 1.7 | 2 | 0.138 |
|  | Midzonal | 3.0 | 3 | 0.554 |
|  | Centrilobular | 6.0 | 1 | **0.003** |
| Case 61 | Portal tract | 3.0 | 2 | 0.138 |
|  | Midzonal | 5.0 | 3 | 0.554 |
|  | Centrilobular | 6.0 | 1 | **0.003** |
| Case 88 | Portal tract | 1.0 | 2 | 0.138 |
|  | Midzonal | 2.0 | 3 | 0.554 |
|  | Centrilobular | 2.3 | 1 | **0.003** |
| Case 91 | Portal tract | 3.3 | 2 | 0.138 |
|  | Midzonal | 3.0 | 3 | 0.554 |
|  | Centrilobular | 2.0 | 1 | **0.003** |
| Case 106 | Portal tract | 5.3 | 2 | 0.138 |
|  | Midzonal | 4.7 | 3 | 0.554 |
|  | Centrilobular | 7.3 | 1 | **0.003** |
| Case 110 | Portal tract | 16.7 | 2 | 0.138 |
|  | Midzonal | 14.7 | 3 | 0.554 |
|  | Centrilobular | 86.3 | 1 | **0.003** |
| Case 117 | Portal tract | 58.3 | 2 | 0.138 |
|  | Midzonal | 3.7 | 3 | 0.554 |
|  | Centrilobular | 1.7 | 1 | **0.003** |
| Total medians | Portal tract | 2.5 | 2 | 0.138 |
|  | Midzonal | 4.0 | 3 | 0.554 |
|  | Centrilobular | 3.5 | 1 | **0.003** |

^Abbreviations: HPF = high power field (400x magnification).^

^*Significant^ *^P^*^-values (^*^P^*^-value <0.05) are in bold.^
